# Biogenesis of lysosome‐related organelles complex‐1 (BORC) regulates late endosomal/lysosomal size through PIKfyve‐dependent phosphatidylinositol‐3,5‐bisphosphate

**DOI:** 10.1111/tra.12679

**Published:** 2019-08-19

**Authors:** Teodor E. Yordanov, Victoria E. B. Hipolito, Gudrun Liebscher, Georg F. Vogel, Taras Stasyk, Caroline Herrmann, Stephan Geley, David Teis, Roberto J. Botelho, Michael W. Hess, Lukas A. Huber

**Affiliations:** ^1^ Division of Cell Biology, Biocenter Medical University of Innsbruck Innsbruck Austria; ^2^ Department of Chemistry and Biology and the Graduate Program in Molecular Science Ryerson University Toronto Ontario Canada; ^3^ Department of Pediatrics I Medical University of Innsbruck Innsbruck Austria; ^4^ Division of Molecular Pathophysiology, Biocenter Medical University of Innsbruck Innsbruck Austria; ^5^ Division of Histology and Embryology Medical University of Innsbruck Innsbruck Austria; ^6^ Austrian Drug Screening Institute, ADSI Innsbruck Austria

**Keywords:** AMP‐activated protein kinase (AMPK), BORC, LAMTOR, late endosome, lysosomal reformation, organelle size, phosphatidylinositol‐3,5‐bisphosphate [PI(3,5)P_2_], PIKfyve

## Abstract

Mechanisms that control lysosomal function are essential for cellular homeostasis. Lysosomes adapt in size and number to cellular needs but little is known about the underlying molecular mechanism. We demonstrate that the late endosomal/lysosomal multimeric BLOC‐1‐related complex (BORC) regulates the size of these organelles via PIKfyve‐dependent phosphatidylinositol‐3,5‐bisphosphate [PI(3,5)P_2_] production. Deletion of the core BORC component Diaskedin led to increased levels of PI(3,5)P_2_, suggesting activation of PIKfyve, and resulted in enhanced lysosomal reformation and subsequent reduction in lysosomal size. This process required AMP‐activated protein kinase (AMPK), a known PIKfyve activator, and was additionally dependent on the late endosomal/lysosomal adaptor, mitogen‐activated protein kinases and mechanistic target of rapamycin activator (LAMTOR/Ragulator) complex. Consistently, in response to glucose limitation, AMPK activated PIKfyve, which induced lysosomal reformation with increased baseline autophagy and was coupled to a decrease in lysosomal size. These adaptations of the late endosomal/lysosomal system reversed under glucose replete growth conditions. In summary, our results demonstrate that BORC regulates lysosomal reformation and size in response to glucose availability.

**Abbreviations**
AMPK: AMP‐activated protein kinase
BLOC‐1: biogenesis of lysosome‐related organelles complex‐1
BORC: BLOC‐1‐related complex
CQ: chloroquine
EEA1: early endosomal antigen 1
IF: immunofluorescence
KO: knock out
LAMP1: lysosome‐associated membrane protein 1
LAMTOR: late endosomal/lysosomal adaptor, MAPK and mTOR activator
MAPK: mitogen‐activated protein kinases
mTOR: mechanistic target of rapamycin
MW: molecular weight
PNS: post‐nuclear supernatant
PtdInsP: phosphoinositides; Phosphatidylinositol 3,5 bisphosphate
SS: steady state
TM: total membranes
WB: Western blotting

## 1 INTRODUCTION

The endocytic system of cells comprises a network of compartments, which are dynamically interacting with each other and with numerous other cellular organelles, such as the Golgi apparatus, the endoplasmic reticulum and the plasma membrane.^1^ The most mature organelle of the endosomal system is the lysosome, it participates in numerous vital cellular processes, such as microbial killing and antigen presentation, detoxification, recycling of metabolic building blocks (amino acids, glucose and cholesterol), apoptosis, cell migration, cancer invasion and metastasis.^2^, ^3^ To adapt to cellular needs and environmental conditions, lysosomes must reform and continuously adapt in size and number. Many lysosomal functions depend on the position of the organelle in the cell. Lysosomes move readily back and forth between the perinuclear region and the cell periphery,^4^ with a majority of them showing a steady state distribution close to the Microtubule Organizing Center (MTOC). The bidirectional movement of lysosomes along microtubules is important for distributing the degradative activity of lysosomes to all regions of the cell, as well as for many other lysosomal functions including antigen presentation, microbial killing, autophagy, metabolic signaling, cell adhesion and migration and tumor invasion and metastasis.^2^, ^3^, ^5^, ^6^, ^7^

To control lysosomal function and signaling capacity, many scaffolding complexes populate the limiting membrane of lysosomes, where they recruit and orchestrate multiple signal transduction cascades.

Two such multimeric endosomal scaffold complexes are the biogenesis of lysosome‐related organelles complex 1 (BLOC‐1) and the late endosomal/lysosomal BLOC‐1‐related complex (BORC). BLOC‐1 comprises the subunits BLOS1, BLOS2, BLOS3, Snapin, Pallidin, Muted, Cappuccino and Dysbindin.^8^, ^9^, ^10^, ^11^ Recent observations suggest that BLOC‐1 may participate in the biogenesis of recycling endosomes by coordinating endosomal tubule generation.^12^

BORC consists only of a subset of BLOC proteins, BLOS1, BLOS2 and Snapin, and additionally employs the specific subunits of this complex KXD1, MEF2BNB, Myrlysin, Lyspersin and Diaskedin.^2^ BORC associates with the cytoplasmic leaflet of the limiting late endosomal/lysosomal membrane, at least partially through an N‐terminal myristoyl group on Myrlysin.^2^ The main function attributed to BORC is the recruitment of the small Arf‐like GTPase Arl8b from the cytoplasm onto the lysosomal membrane, which promotes the kinesin‐dependent movement of lysosomes toward the cell periphery along microtubule tracks.^2^, ^13^ Consistently, BORC deficient cells accumulate lysosomes in the perinuclear region and exhibit reduced spreading and motility. Recently, we demonstrated that the C‐terminus of the BORC subunit Lyspersin is essential and sufficient for BORC‐dependent recruitment of Arl8b to lysosomes.^14^ In addition, we established Lyspersin as the linker between BORC and LAMTOR/Ragulator complexes and identified LAMTOR/Ragulator as a negative regulator of BORC‐ and Arl8b‐dependent lysosomal transport to the cell periphery. Importantly, growth factor stimulation as well as amino acid availability control lysosomal positioning through a LAMTOR/Ragulator dependent, but mammalian target of rapamycin complex 1 (mTORC1)‐independent pathway.^14^, ^15^ The BORC complex together with Arl8b is required to allow the movement of a subset of lysosomes to the periphery of the cell.^2^, ^16^

Here, we present evidence that BORC is also involved in the control of late endosomal/lysosomal size, which is mediated via regulation of phosphatidylinositol‐3,5‐bisphosphate [PI(3,5)P_2_] levels. Cells lacking the core BORC components, Diaskedin or Myrlysin had smaller late endosomes/lysosomes compared to their wild type (WT) counterparts. In contrast, deletion of a third core BORC component Lyspersin did not affect the size of these organelles, pointing toward a specific function of Diaskedin and Myrlysin in this process. This size difference was retained even under conditions, where fundamental lysosomal properties such as pH were compromised. The morphological changes of the late endosomes/lysosomes in BORC deficient cells were caused by enhanced lysosomal reformation—a process, directly linked to PIKfyve function. Recently, it was reported that inhibiting PIKfyve impairs generation of terminal lysosomes, since PIKfyve activity regulates extensive membrane remodeling that initiates reformation of lysosomes from acidic and hydrolase‐active, enlarged endolysosomes.^17^, ^18^

Upstream from PIKfyve, we have identified AMPK as key activator. Endosomal size regulation was correlated with cellular energy status and AMPK activation. Under glucose starvation, a well‐established activator of AMPK signaling,^19^, ^20^ a significant reduction of late endosomal size in WT cells was achieved, which was morphologically undistinguishable from the Diaskedin deletion mutant, bearing intrinsically activated AMPK signaling. Organelle size could then be reverted to the steady state dimensions upon glucose restimulation, pointing toward an energy sensing involvement of the BORC complex. In addition, the LAMTOR/Ragulator complex was found as a modulator of this process. LAMTOR/Ragulator deficient cells phenocopy BORC deletion mutants in terms of endosomal/lysosomal size and abundance. Overall, our data suggest that BORC controls the size of late endosomes and lysosomes in addition to its established role in controlling lysosomal transport. Finally, baseline autophagy in Diaskedin and Myrlysin deletion mutants was also significantly upregulated. We could link this phenotype to the decrease of endosomal/lysosomal size, as both processes are ultimately regulated by PI(3,5)P_2_ production.

## 2 RESULTS

### 2.1 Deletion of the BORC component Diaskedin reduces the size of lysosomes

**Figure 1:**
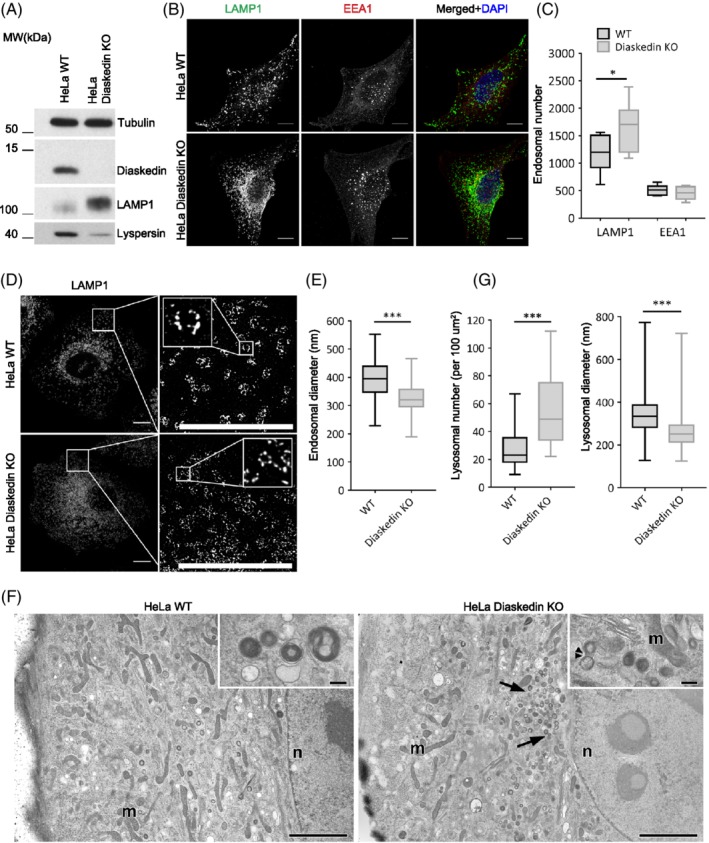
Deletion of Diaskedin causes reduction of late endosomal size in HeLa. (A) WB of cell lysates of HeLa WT and Diaskedin KO cells. (B) Single plane images of Z‐stacks of WT HeLa and Diaskedin KO, stained with the late endosomal marker LAMP1 and the early endosomal marker EEA1. Scale bars: 10 μm. (C) Frequency of endosomes in WT and Diaskedin KO HeLa based on analyses of Z‐stack confocal images. Total endosomal count was acquired using automated counting algorithm in Imaris from 10 cells per genotype. Data presented as median value with the box representing the 25th and 75th percentile, and the whiskers—the minimal and maximal values. Unpaired Student's *t* test was performed between WT and KO samples for each endosomal population (*P ≤ .05; **P ≤ .01; ***P ≤ .001). (D) Super resolution STED images of WT HeLa and Diaskedin KO. Inserts show magnification of the selected areas. Scale bars: 10um. (E) Endosomal diameters in WT and Diaskedin KO HeLa based on measurements from superresolution STED images. Manual size measurement of at least 100 endosomes from at least six cells per genotype was made. Data presented as median value with the box representing the 25th and 75th percentile, and the whiskers—the minimal and maximal values. Unpaired Student's *t* test was performed between WT and KO samples (*P ≤ .05; **P ≤ .01; ***P ≤ .001). (F) Electron microscopy (EM) of cryofixed HeLa WT and Diaskedin KO cells with perinuclear clustering of lysosomes (arrows) in Diaskedin KO cells (m: mitochondria, n: nucleus). Scale bars: 2 μm. Inserts show high magnification views of lysosomes, one of which with a putative constriction site (double arrow‐head) in KO cells. Scale bars: 200 nm. (G) Frequency of lysosomes per 100 square‐micron cytoplasm and lysosomal size in WT and Diaskedin KO HeLa cells as measured on EM images (>550 lysosomes from >25 cells per genotype). Data presented as median value with the box representing the 25th and 75th percentile, and the whiskers—the minimal and maximal values. Unpaired Student's *t* test was performed between WT and KO (*P ≤ .05; **P ≤ .01; ***P ≤ .001)

Recently, we and others^14^, ^15^ have demonstrated that the BORC subunit Lyspersin is specifically required for establishing the interaction between BORC and LAMTOR/Ragulator on the surface of late endosomes/lysosomes. Therefore, we hypothesized that other BORC subunits might as well fulfill dedicated functions. To test this assumption, we first selected Diaskedin for further analysis since it was shown to interact in equimolar ratios with Myrlysin, the proposed membrane anchor of the complex.^21^ Upon CRISPR/Cas9 mediated knock‐out (KO) of Diaskedin in HeLa cells, we noticed that the protein levels of the BORC subunit, Lyspersin, were downregulated, whereas the protein levels of the late endosomal marker lysosome‐associated membrane protein 1 (LAMP1) were upregulated (Figure 1A). Consistently, the morphology of the late endosomal/lysosomal system was severely affected in Diaskedin deficient (KO) cells. The total number of LAMP1 positive late endosomes/lysosomes was significantly increased compared with WT cells (Figure 1B,C) and those organelles typically clustered in the perinuclear region (Figure 1B,F), which resembled the phenotype of Lyspersin depleted cells.^14^ The number of early endosomal antigen 1 (EEA1) positive early endosomes was comparable in Diaskedin KO and WT cells (Figure 1B,C). Super resolution stimulated emission depletion (STED) microscopy revealed that the diameter of individual LAMP1 positive late endosomes/lysosomes was significantly reduced in Diaskedin KO cells, compared with WT HeLa cells (Figure 1D,E). Additionally, morphometry from electron microscopy (EM) images (Figure 1F) of high‐pressure frozen HeLa cells further confirmed an increased frequency of lysosomes, their perinuclear accumulation, as well as their smaller diameter upon Diaskedin deletion (Figure 1F,G).

### 2.2 Reduction of endosomal size upon deletion of different BORC components in HT1080 cells

**Figure 2:**
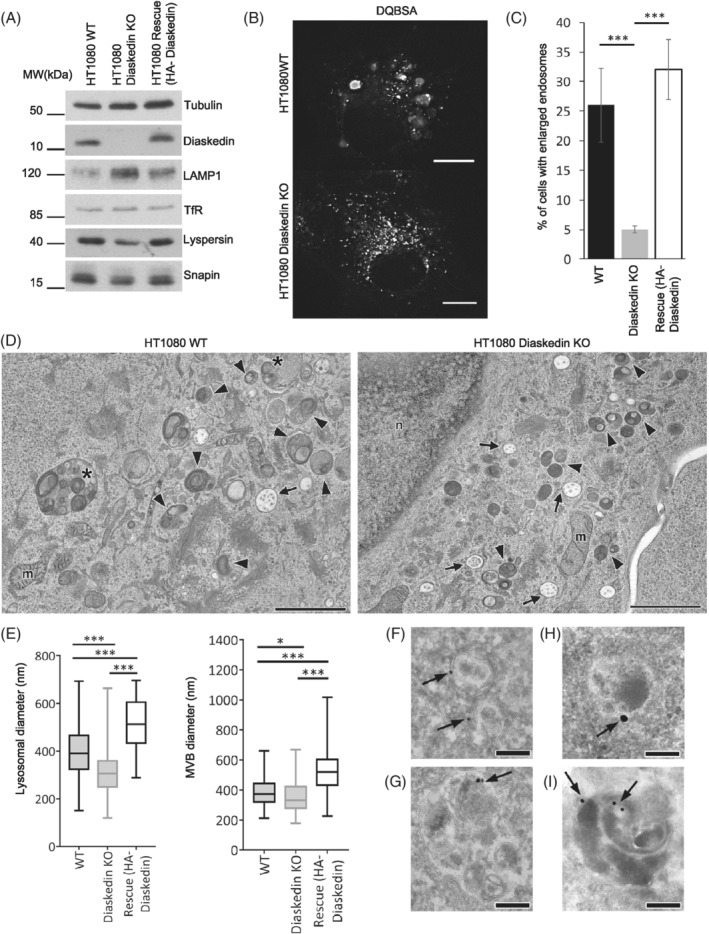
Formation of characteristic enlarged late endosomes and size of multivesicular bodies (MVBs)/lysosomes in HT1080 is directly dependent on Diaskedin. (A) Representative WB of total protein levels in WT, Diaskedin KO or Diaskedin KO cells, stably expressing WT HA‐tagged Diaskedin (Rescue). (B) Still images taken from live‐cell movies of WT and Diaskedin KO HT1080 cells, fed with fluorescently labeled DQBSA. Snapshots taken from movie S1. Scale bars: 10 μm. (C) Quantification of the percentage of WT, Diaskedin KO and Rescue cells that display HT1080 characteristic enlarged late endosomes. Cells were counted from at least three biological replicates with over 100 cells per genotype pro replica. Data presented as mean values ± SD. Unpaired Student's *t* test was performed between all genotypes (*P ≤ .05; **P ≤ .01; ***P ≤ .001). (D) Electron microscopy of cryofixed WT and Diaskedin KO HT1080 cells illustrating differences in size and morphology throughout all kinds of endo−/lysosomal compartments under steady state (arrows: MVBs; arrowheads: lysosomes; asterisks: enlarged late endosomes; m: mitochondria; n: nucleus). Scale bars: 2 μm. (E) Diameters of normal‐sized (≤700 nm) lysosomes and MVBs from WT, Diaskedin KO and Rescue HT1080 cell lines as measured by EM. At least 200 lysosomes or 120 MVBs, respectively, per genotype were measured. Data presented as median value with the box representing the 25th and 75th percentile, and the whiskers—the minimal and maximal values. Unpaired Student's *t* test was performed between all genotypes (*P ≤ .05; **P ≤ .01; ***P ≤ .001). (F‐I) Immunogold electron microscopy of Diaskedin KO cells, stably expressing HA‐tagged WT Diaskedin (Rescue). Anti‐HA label localizes predominantly at the limiting membrane of MVBs/late endosomes (F,G,H), lysosomes (I); Tokuyasu‐cryosection technique, scale bar: 200 nm

To corroborate these observations in another cell line, we generated a Diaskedin KO in the fibrosarcoma cells HT1080, together with the respective rescue cell line by re‐expressing HA‐tagged WT Diaskedin (Figure 2A). The loss of Diaskedin in these cells was also accompanied by LAMP1 upregulation and a slight downregulation of Lyspersin (Figure 2A). Interestingly, roughly 25% of WT HT1080 cells harbor already under standard growth conditions a population of considerably enlarged late endocytic compartments (often >2 μm in diameter; Figure 2B‐D). These oversized endocytic compartments were positive for several marker proteins of late endosomes/lysosomes (LAMP1, CD63 and Cathepsin D), but were devoid of EEA1 and M6PR (Supplementary Figure S1A‐C). The functional properties of the oversized compartments, such as luminal pH, accumulation of fluid phase markers and degradative capacity also appeared comparable to the other, normal‐sized late endosomes/lysosomes in the same cells (Figure 2B and Movie S1). Thus, we concluded that those oversized endocytic compartments were catabolically active late endosomes/lysosomes and we refer to them as “enlarged late endosomes” hereafter. Their exaggerated diameter greatly facilitated light microscopic monitoring of organelle alterations. Importantly, upon Diaskedin deletion, there was a lower frequency of cells with enlarged endosomes (Figure 2C). In the Diaskedin HT1080 rescue cell line, re‐expressing HA‐tagged WT Diaskedin, the frequency of enlarged late endosomes was similar to WT HT1080 cells (Figure 2A,C).

By applying high‐pressure freezing in combination with EM we determined alterations in size and morphology throughout the endocytic compartments in HT1080 WT and the corresponding Diaskedin KO cells under steady state conditions (Figure 2D). In WT cells lysosomes and multivesicular bodies were generally larger than in Diaskedin KOs (Figure 2D,E). Some WT cells also contained enlarged late endosomes (Figure 2D asterisks). These enlarged endosomes were excluded from ultrastructural morphometry and we focused on the precise measurements of normal‐sized organelles with diameters ranging from approximately 150 to 700 nm. Diaskedin KO cells were characterized by numerous small, dense lysosomes, that were rare in WT cells. In the Diaskedin deficient cell line, which re‐expressed HA‐Diaskedin, the size of late endosomes/lysosomes was similar to WT cells and the anti‐HA label localized predominantly at the limiting membrane of multivesicular bodies/late endosomes (Figure 2F‐H) and lysosomes (Figure 2I). Overall, these results suggested that Diaskedin localizes to late endosomes/lysosomes and controls their size and abundance.

### 2.3 Deletions of Diaskedin and Myrlysin but not Lyspersin abolish the formation of characteristic enlarged endosomes in HT1080

**Figure 3:**
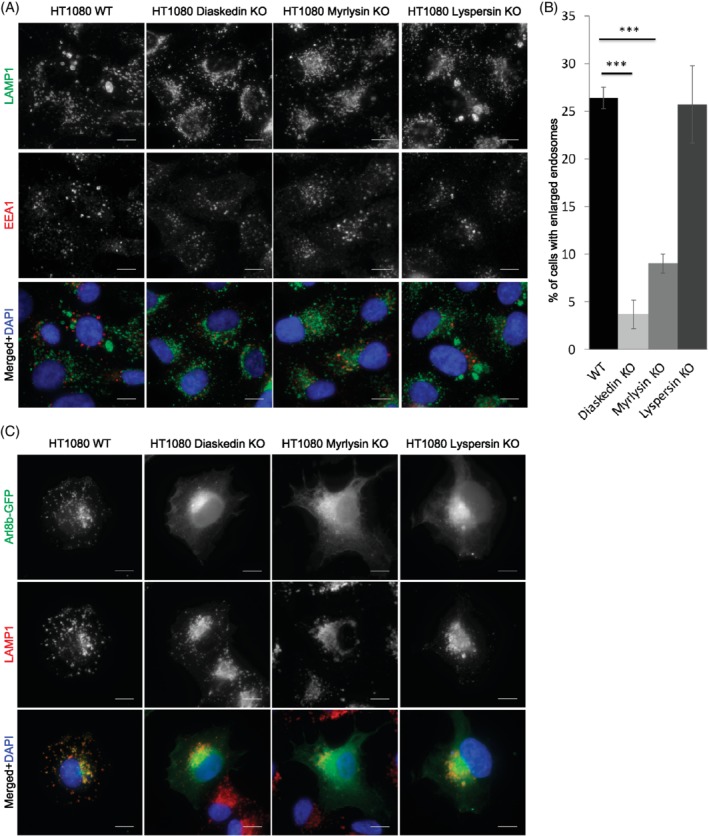
Reduction of characteristic enlarged late endosomes upon deletion of Diaskedin and Myrlysin but not Lyspersin in HT1080 cells. (A) IF of WT, Diaskedin KO, Myrlysin KO and Lyspersin KO HT1080 cells, stained with the late endosomal marker LAMP1 (green) and the early endosomal marker EEA1 (red). Scale bars: 10um. (B) Quantification of the percentage of cells that display HT1080 characteristic enlarged late endosomes. Cells were counted from at least three biological replicates with over 100 cells per genotype pro replica. Data presented as mean values ± SD. Unpaired Student's *t* test was performed between each KO and the WT control (*P ≤ .05; **P ≤ .01; ***P ≤ .001). (C) IF of WT, Diaskedin KO, Myrlysin KO and Lyspersin KO HT1080 cells, transiently transfected with Arl8b‐GFP (green) and costained with the late endosomal marker LAMP1 (red). Scale bars: 10um

We next asked if other BORC components were also required to control the size of late endosomes/lysosomes similar to Diaskedin (Figure 2C and Figure 3A,B). Therefore, we also deleted Myrlysin, the membrane anchor of the BORC, and Lyspersin. In all three BORC mutants (Diaskedin, Myrlysin and Lyspersin), the small GTPase Arl8b mislocalized from late endosomes to the cytoplasm—a well described BORC deletion phenotype (Figure 3C). Deletion of Myrlysin also reduced the number of enlarged late endosomes and phenocopied Diaskedin KO cells. Yet, Lyspersin deficient late endosomes/lysosomes were indistinguishable from their WT counterparts in this aspect (Figure 3A,B). Additionally, the amount of BORC components bound to membranes in the cell, was generally altered upon Diaskedin and Myrlysin but not Lyspersin deletion (Supplementary Figure S1D,E). This led us to conclude that the size of late endosomes/lysosomes was specifically controlled by Diaskedin and Myrlysin, but not to all BORC components alike and was independent of Arl8b. The enlarged late endosomes observed in HT1080 WT cells were reminiscent of endolysosomes^17^, ^22^ in cells treated with the lysosomotropic antimalarial drug chloroquine (CQ)^22^ that prevents endosomal acidification. HT1080 WT cells exposed to CQ for 2 hours displayed an increased number of enlarged late endosomes compared to control cells at steady state (Supplementary Figure S2A,B). This increase in size was suppressed by Diaskedin or Myrlysin deletion but not by Lyspersin deletion. Blocking the V‐ATPase with bafilomycin A had a comparable effect (Supplementary Figure S2C). Stimulation of macropinocytosis through phorbol 12‐myristate 13‐acetate (PMA) administration enhanced the formation of enlarged endosomes more efficiently in WT cells and was dependent on Diaskedin (Supplementary Figure S2C). Inhibition of mechanistic target of rapamycin (mTOR) through rapamycin treatment or amino acid starvation had no influence on the cell population with enlarged late endosomes (Supplementary Figure S2C). Taking these findings together, we concluded that Diaskedin and Myrlysin are required for the formation of enlarged late endosomes in HT1080 cells. Alternatively, they may negatively regulate clearance of these organelles via lysosomal reformation.

### 2.4 PI(3,5)P_2_ levels regulate late endosomal size downstream of BORC

**Figure 4:**
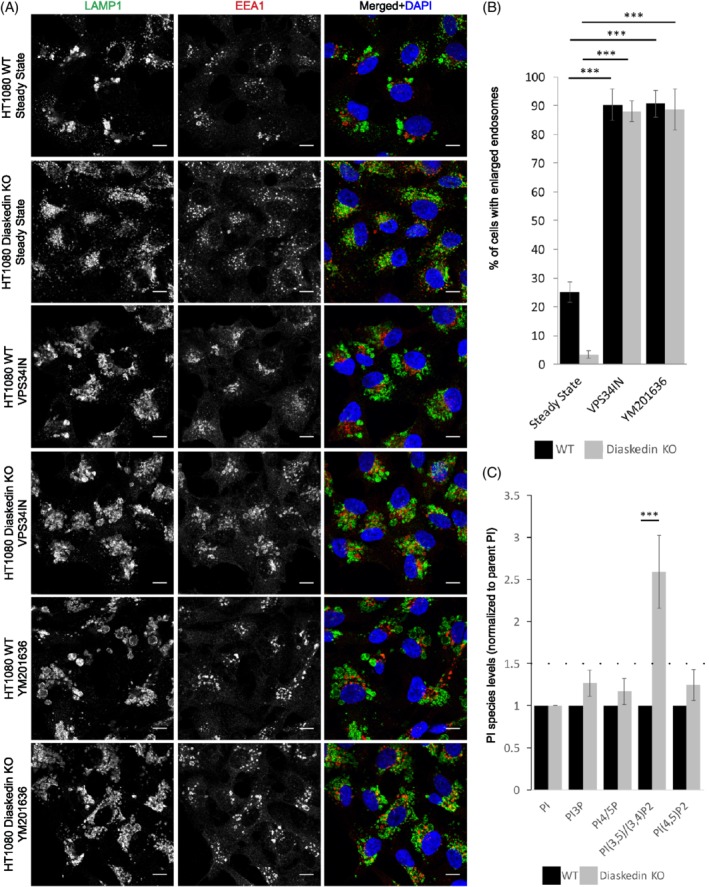
Inhibition of PI(3,5)P_2_ synthesis induces the formation of characteristic enlarged late endosomes in HT1080 cells. (A) Confocal images of WT and Diaskedin KO HT1080 cells in steady state and treated with either 2.5uM VPS34IN or 800 nM YM201636 for 2 hours and stained with LAMP1 (green) and EEA1 (red). Scale bars: 10 μm. (B) Quantification of the percentage of cells that display HT1080 characteristic enlarged late endosomes either untreated in steady state or with inhibitor treatments. Cells were counted from at least three biological replicates with over 100 cells per genotype and tested condition pro replica. Data presented as mean values ± SD. Unpaired Student's *t* test was performed for every genotype in the tested conditions (*P ≤ .05; **P ≤ .01; ***P ≤ .001). (C) Comparison of the abundancy of the indicated PtdInsPs in WT and Diaskedin KO HT1080 cells labeled with ^3^H‐*myo*‐inositol. Data presented as normalized (to total PI) mean values ± SD. Unpaired Student's *t* test was performed between WT and Diaskedin KO for every PtdInsP species where a difference of over 1.5x‐fold (dotted line) was observed from at least three independent biological replicates (*P ≤ .05; **P ≤ .01; ***P ≤ .001)

Having established that specific BORC subunits are required to regulate late endosomal/lysosomal size, we sought out to characterize the underlying molecular mechanism. One candidate was size regulation via the phosphatidylinositol phosphate species PI(3,5)P_2_. PI(3,5)P_2_ is generated from phosphatidylinositol *3*‐phosphate [PI(3)P] by the lipid kinase PIKfyve and has been linked to the regulation of endosomal size and number.^17^, ^18^, ^23^ The lipid kinase PIKfyve phosphorylates PI(3)P to generate PI(3,5)P_2_ on late endosomes/lysosomes. PI(3,5)P_2_ plays an important role in late endosomal biogenesis. The reduction of PI(3,5)P_2_ levels leads to the enlargement of late endosomes/lysosomes and contributes to a variety of diseases.^18^, ^23^, ^24^, ^25^ To address the link between BORC and PI(3,5)P_2_ levels on endosomal size regulation, we used two established inhibitors, which block PI(3,5)P_2_ production; either by directly inhibiting PIKfyve with YM201636, or by blocking the upstream production of PI(3)P with VPS34‐IN1 (Supplementary Figure S3A) and measured their effects on the frequency of enlarged late endosomes/lysosomes in HT1080 cells (Figure 4A,B). Both inhibitors increased the frequency of enlarged endosomes/lysosomes, with the effect of YM201636 being more specific to late endosomes/lysosomes, whereas VPS34‐IN1 also induced inflation of early endosomes (Figure 4A). Upon inhibition of PI(3,5)P_2_, almost all Diaskedin KO cells displayed enlarged endosomes, similar to WT cells under the same condition. Similar results were obtained in HeLa cells treated with the above described inhibitors for 1 hour, and subsequently washed and recovered in inhibitor free medium for 1 hour (Supplementary Figure S3B,C). Based on these results we speculated that loss of Diasekdin resulted in hyperactive PIKfyve, which generated higher PI(3,5)P_2_ levels leading to the reduction of late endosomal/lysosomal size.

To directly test this idea, we determined the changes in phosphoinositides (PtdInsP) species in Diaskedin deficient HT1080 cells compared to their WT counterparts. Therefore, HT1080 cells were incubated with ^3^H‐*myo*‐inositol to label PtdInsPs, followed by lipid extraction and deacylation, and the amount of PtdInsP species was compared to total phosphatidylinositol by flow scintillation. Most of the major PtdInsP species were similar between the WT and Diaskedin KO cells (Figure 4C). However, levels of PI(3,5)P_2_ and/or PI(3,4)P_2_, which for technical reasons are difficult to separate from each other by high performance liquid chromatography (HPLC), were higher in Diaskedin KO compared to WT cells (Figure 4C). To discriminate between PI(3,5)P_2_ and PI(3,4)P_2_, additional experiments were performed. For this purpose, cells were pretreated with the PIKfyve inhibitor Apilimod (Supplementary Figure S3A), which completely abolished the signal from this peak and caused subsequent accumulation of the precursor PI(3)P (Supplementary Figure S3,D). This indicated that the significant increase, detected in steady state cells, was indeed caused by an increase of PI(3,5)P_2_. Most importantly, these experiments indicated that Diaskedin deletion resulted in a higher PI(3,5)P_2_ production, which is indicative of an increased activity of PIKfyve—the only known lipid kinase to phosphorylate PI(3)P to PI(3,5)P_2_. Thus, the smaller size of endosomes in Diaskedin KO cells correlated with increased levels of PI(3,5)P_2_, suggesting an inhibitory role for the BORC complex toward PIKfyve activity and PI(3,5)P_2_ production.

### 2.5 Diaskedin KO enhances tubule formation and lysosomal reformation

**Figure 5:**
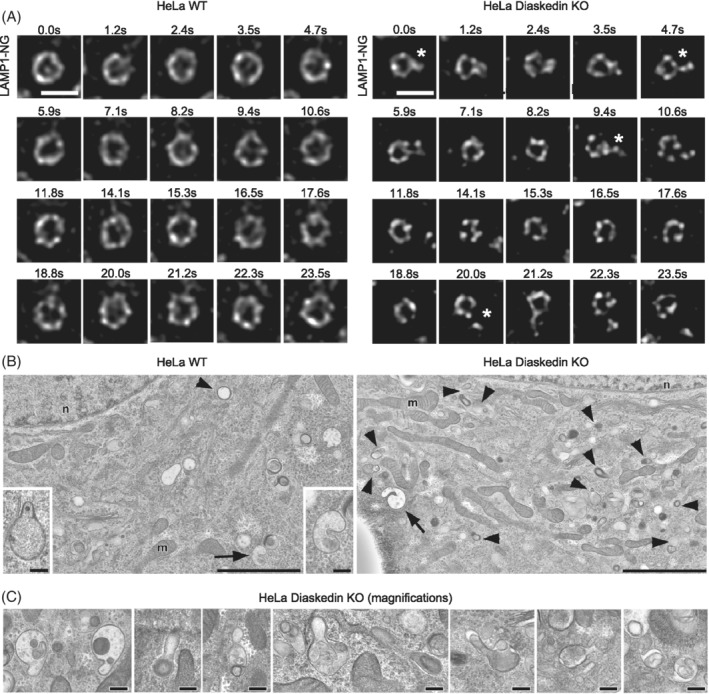
Diaskedin KO leads to hyperactivated tubule formation of late endosomes/lysosomes. (A) Still images from movies S2 and S3 depicting WT and Diaskedin KO HeLa cells, stably expressing LAMP1‐NeonGreen and imaged over a period of 23.5 seconds with a super resolution STED microscope. The indicated stars mark the frames in which tubules are emerging from late endosomes/lysosomes. Scale bars: 500 nm. (B) EM reveals in Diaskedin KO HeLa cells (right) observed at steady state numerous elongated profiles of late endosomes (arrows) and lysosomes (arrow‐heads) presumably reflecting tubule formation and/or fission; these features are almost completely missing in WT HeLa cells (left). Scale bars: 2 μm in overviews, 200 nm in inserts. (C) Diaskedin KO HeLa cells at steady state show in 80 nm‐ultrathin EM‐sections late endosomes, lysosomes and autophago(lyso)somes with snapshot profiles suggestive of tubule formation and/or fission, which resemble the patterns of AA/FBS starvation (see also Figure 10D). Scale bars: 200 nm

PI(3,5)P_2_ can drive the reformation of terminal lysosomes from acidic, hydrolase‐active enlarged endolysosomes^17^ and thereby promotes lysosome fission to maintain lysosome size and number.^18^ Since PI(3,5)P_2_ levels were increased in Diaskedin KO cells, we assumed that excessive lysosomal reformation occurs in these cells. This would result in unrestrained tubule formation and/or fission from late endosomes/lysosomes, resulting in ongoing lysosomal reformation and ultimately leading to the formation of smaller late endosomes/lysosomes. To test our assumptions in live cells over time, we generated LAMP1‐NeonGreen expressing HeLa WT and Diaskedin KO cell lines and analyzed tubule formation from late endosomes/lysosomes using live‐cell STED microscopy (Movies S2 and S3). We were able to follow individual LAMP1‐NeonGreen positive endosomes for about 23 seconds. During this time, LAMP1‐NeonGreen positive endosomes in WT HeLa cells remained relatively stable in size and shape and only a small portion of them displayed emerging tubules (2.6% based on over 100 endosomes in the first frames of seven independent movies) (Figure 5A). In the Diaskedin KO HeLa cells, the diameter of LAMP1‐NeonGreen positive endosomes was smaller and they displayed repetitive tubulation events (10.3% based on over 100 endosomes in the first frames of seven independent movies), pointing to enhanced lysosomal reformation. Ultrastructural analysis using transmission electron microscopy (TEM) from high‐pressure frozen samples at steady state (Figure 5B,C) supported these data. Diaskedin KO HeLa cells displayed numerous elongated profiles of late endosomes/lysosomes consistent with tubule formation and/or fission. Similar morphological features were hardly detected in WT HeLa cells. From the analysis of cross sections of approximately 200 cells per genotype, we roughly estimated the frequency of these tubulation events: EM sections from WT cells showed in total three cells with one singular elongated organelle profile, as compared to 45 cells with numerous (>10) elongated/tubule forming organelles per cell in Diaskedin KO.

In a complementary approach, transient inhibition and subsequent reactivation of PIKfyve was used to boost tubule formation and/or fission events in HT1080 cells. In this experimental setup, WT and Diaskedin KO HT1080 cells were first treated with YM201636, then the inhibitor was washed‐out and the cells left to recover in an inhibitor free medium. During the recovery phase, cells were high‐pressure‐frozen and analyzed by EM in combination with tomography. Under these extreme conditions, both cell lines displayed enhanced tubule formation of endolysomal compartments. However, the process was still enhanced in Diaskedin KO cells, with more, though smaller tubules appearing in the Diaskedin KO cells, compared with their WT counterparts (Movies S4 and S5). These experiments indicated that activation of PIKfyve and the resulting increase in PI(3,5)P_2_ levels in Diaskedin KO indeed translated into more unrestrained lysosomal reformation and fission, which ultimately led to the observed decrease of late endosomal size.

### 2.6 AMPK activity modulates BORC's control over endosomal size

Next, we examined how BORC coupled to lysosomal signaling to restrain lysosomal reformation via PIKfyve. Several signaling pathways are recruited to late endosomes/lysosomes, by the LAMTOR/Ragulator complex, including mitogen‐activated protein kinase (MAPK), AMP‐activated protein kinase (AMPK) and mTORC1.^26^, ^27^, ^28^, ^29^, ^30^, ^31^ So far there have been only few reports on how PIKfyve is regulated, but two major kinases, namely AMPK^32^ and AKT^33^ have been reported to phosphorylate and thereby activate PIKfyve. Diaskedin deletion mutants have intrinsically more active AMPK and ERK signaling under most growth conditions, whereas AKT and mTORC1 (pS6K, pS6) signaling were largely unchanged compared to WT cells (Supplementary Figure S4A). Hence in Diaskedin deficient cells, AMPK signaling from the putative BORC localization at late endosomes might lead to higher PIKfyve activation.

**Figure 6:**
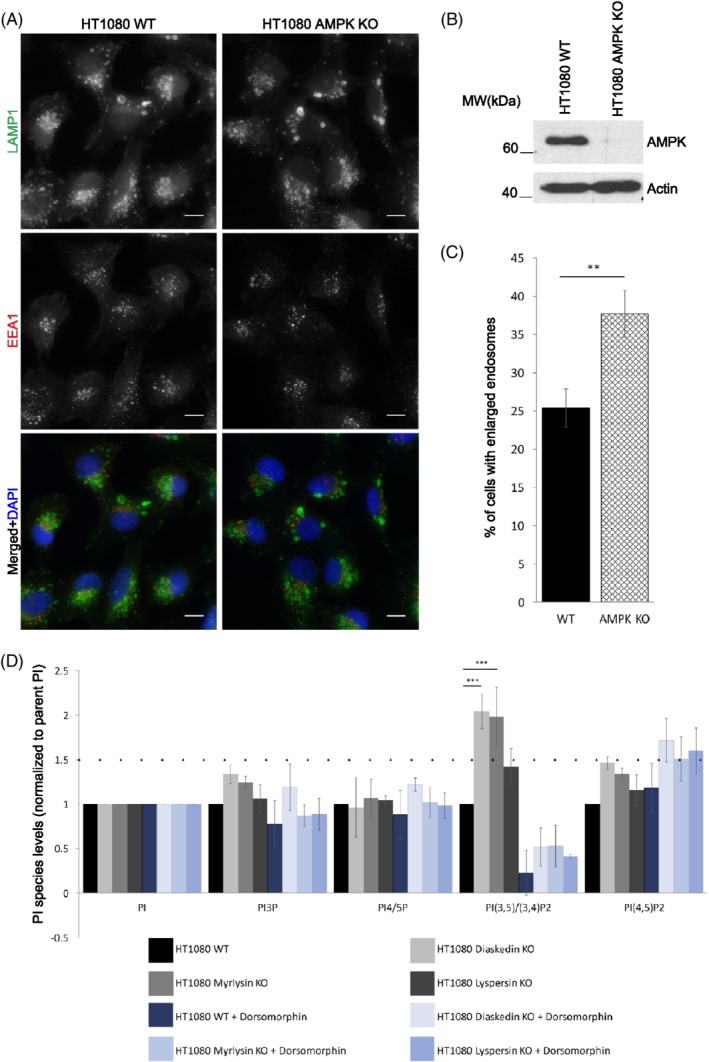
Deletion of AMPK leads to increased formation of characteristic enlarged late endosomes in HT1080 cells. (A) IF of WT and AMPK KO (subunit Alpha) HT1080 cells, stained with the late endosomal marker LAMP1 (green) and the early endosomal marker EEA1 (red). Scale bars: 10 μm. (B) WB analysis of WT and AMPK KO HT1080 cells. (C) Quantification of the percentage of cells that display HT1080 characteristic enlarged late endosomes. Cells were counted from at least three biological replicates with over 100 cells per genotype pro replica. Data presented as mean values ± SD. Unpaired Student's *t* test was performed between each genotype (*P ≤ .05; **P ≤ .01; ***P ≤ .001). (D) WT and AMPK KO HT1080 cells were incubated with ^3^H‐*myo*‐ inositol and the indicated PtdInsP species and their abundancy was compared in either steady state or upon Dorsomorphin addition. Data presented as normalized (to total PI) mean values ± SD. Unpaired Student's *t* test was performed between WT and Diaskedin KO for every PtdInsP species where a difference of over 1.5x‐fold (dotted line) was observed from at least three independent biological replicates (*P ≤ .05; **P ≤ .01; ***P ≤ .001)

To directly test how AMPK signaling might control late endosomal/lyosomal size, we generated a CRISPR/Cas9 mediated KO of the alpha subunit of AMPK in HT1080 cells (Figure 6A‐C). Upon AMPK deletion, significantly more cells displayed enlarged late endosomes, indicating that AMPK modulated this phenotype. Therefore, we next asked whether AMPK signaling could help to explain the function of Diaskedin and Myrlysin in endosomal size regulation via enhanced PIKfyve activity and the resulting increase in PI(3,5)P_2_. To address this assumption, we performed PtdInsP lipid measurements in all HT1080 BORC deficient cell lines either in steady state or upon the addition of the specific AMPK inhibitor Dorsomorphin (Figure 6D). Here, we noticed that not only Diaskedin KO, but also deletion of the BORC‐anchor Myrlysin caused significant accumulation of PI(3,5)P_2_. Lyspersin mutants showed only a mild increase. Importantly, the increase in PI(3,5)P_2_ production in Diaskedin and Myrlysin deletion mutants could be significantly inhibited by the treatment of the cells with Dorsomorphin. Consistently, Diaskedin (Supplementary Figure S4A) and Myrlysin KOs had increased activation of AMPK, whereas Lyspersin deletion mutants did not (data not shown). Taken together, these experiments link the BORC subunits Diaskedin and Myrlysin to AMPK signaling, which in turn activates PIKfyve to regulate late endosomal/lysosomal size regulation via PI(3,5)P_2_ production.

### 2.7 Contribution of the LAMTOR/Ragulator complex

**Figure 7:**
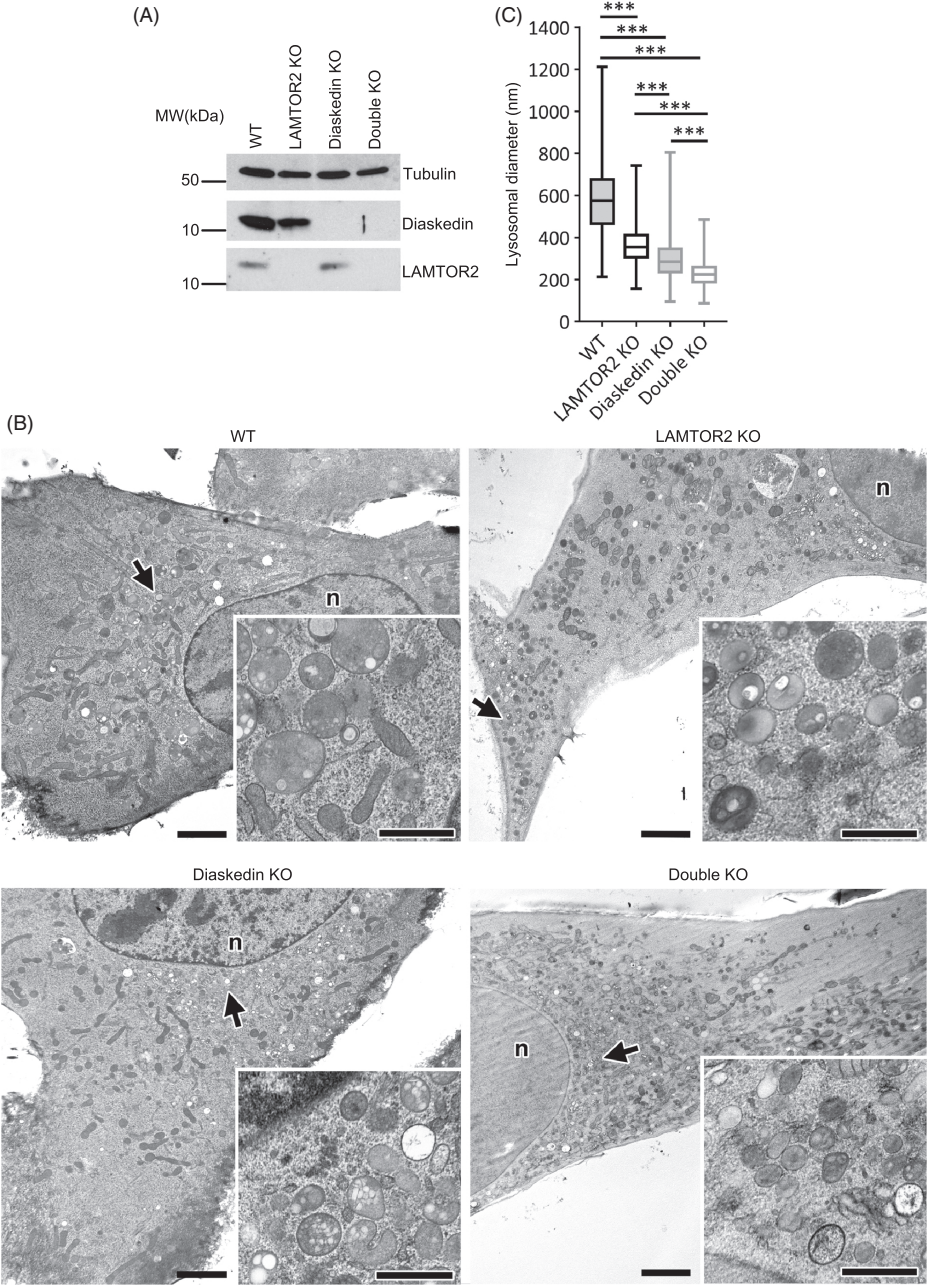
Endosomal phenotypes in LAMTOR/Ragulator KO, BORC KO and LAMTOR/Ragulator‐BORC Double KO MEFs. (A) Representative WB of total protein levels in WT (LAMTOR2 f/−), LAMTOR2 KO (−/−), Diaskedin KO and Double KO MEFs (LAMTOR2 −/− MEFs subjected to Diaskedin KO). (B) WT MEFs (LAMTOR2 f/−) show a canonical phenotype regarding lysosomal size and distribution. By contrast, LAMTOR2 KO (−/−) MEFs contain smaller lysosomes, most of which are typically translocated to the cell periphery. Diaskedin KO MEFs are characterized by perinuclear clustering of lysosomes that are even smaller than those of LAMTOR2 KO (−/−) MEFs. Double KO MEFs, (LAMTOR2 −/− MEFs subjected to Diaskedin KO), also show distinct perinuclear clustering of lysosomes, that are again clearly smaller than those of the other MEF lines investigated here. Arrows mark the sites of the (partly rotated/mirrored) inserts. Scale bars: 2 μm in overviews, 1 μm in inserts (n: nucleus). (C) Lysosomal diameters in the indicated MEF cell lines as measured on EM images. Over 600 lysosomes from over 25 cells per genotype were measured. Data presented as median value with the box representing the 25th and 75th percentile, and the whiskers—the minimal and maximal values. Unpaired Student's *t* test was performed for every genotype (*P ≤ .05; **P ≤ .01; ***P ≤ .001)

The LAMTOR/Ragulator complex is a major player in the regulation of late endosomal/lysosomal signaling as it recruits and facilitates MAPK, AMPK and mTORC1 activation on late endosomes/lysosomes.^30^, ^34^ Since BORC and the LAMTOR/Ragulator complex interact on late endosomes/lysosomes,^14^, ^15^ we addressed how their interaction contributed to endosomal size regulation. Therefore, we generated CRISPR/Cas9 KO cells of Diaskedin in mouse embryonic fibroblasts (MEFs) bearing either a single WT allele (f/−, control) or complete LAMTOR2 deletion (−/−, LAMTOR2KO) as previously established^27^ (Figure 7A). Deletion of LAMTOR2 is sufficient to cause a rapid disassembly and degradation of the entire LAMTOR complex.^14^, ^35^ By high‐pressure freezing in combination with EM, we performed extensive morphometry analysis of late endosomal/lysosomal compartments in these MEFs (Figure 7B,C). MEFs bearing a Diaskedin deletion had significantly smaller lysosomes compared to their control counterparts and clustered in the perinuclear region (Figure 7B,C). Hence, loss of Diaskedin triggered the formation of smaller late endosomes/lysosomes in HeLa (Figure 1), HT1080 (Figure 2) and in MEF cells (Figure 7). Consistent with our previous observations, late endosomes/lysosomes in LAMTOR2 deficient MEFs were translocated toward the periphery of the cell^27^ and were also smaller,^36^ albeit the significant size reduction was not as dramatic as in Diaskedin deletion mutants. When both LAMTOR2 and Diaskedin were deleted (ie, Double KO), late endosomes/lysosomes were clustered in the perinuclear region and their size was significantly smaller than in any of the single KO MEFs or WT cells (Figure 7B,C), indicating an additive effect of BORC and its interacting LAMTOR/Ragulator complex in endosomal size regulation.

### 2.8 Reduction of endosomal size upon glucose starvation

AMPK is a major sensor of the cellular energy status in eukaryotic cells,^37^ responding to low energy status by switching off anabolic pathways and upregulating catabolic processes. Recently, it was demonstrated that different intensities of AMPK stimulation trigger activation of specific subcellular pools, resulting in phosphorylation of distinct downstream targets. Under conditions of glucose starvation that do not elevate cellular adenosine monophosphate: ADP or adenosine diphosphate:ATP ratios, only lysosomal AMPK is activated and exclusively through the lysosomal pathway.^38^

**Figure 8:**
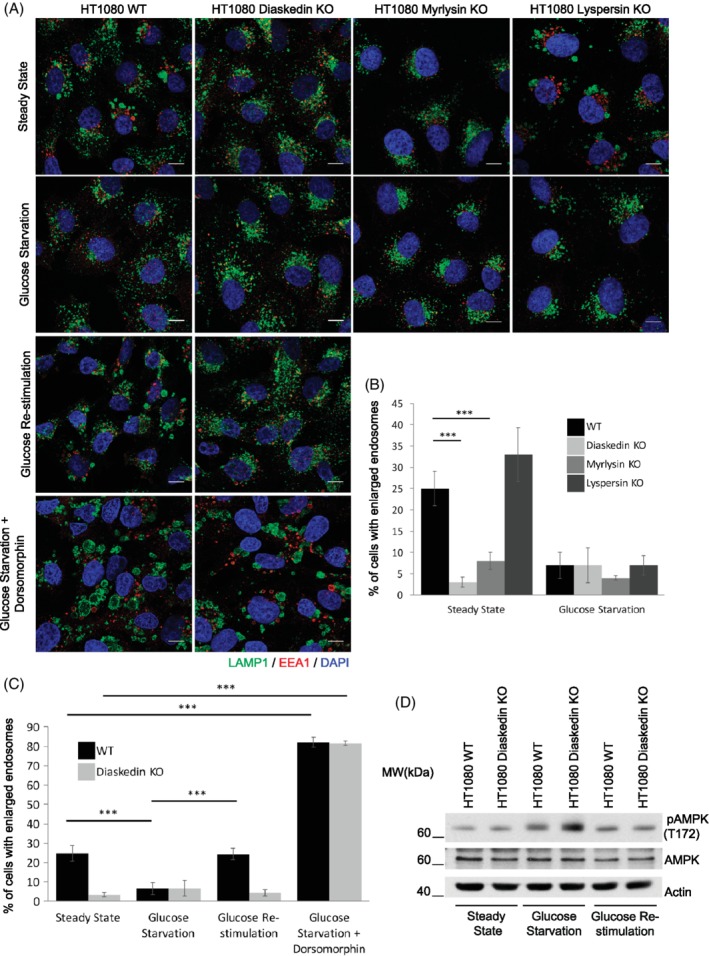
Reduction of characteristic enlarged late endosomes in HT1080 cells upon energy stress. (A) Confocal images of WT, Diaskedin KO, Myrlysin KO and Lyspersin KO HT1080 cells in steady state or upon 12 hours glucose starvation. Additionally, WT and Diaskedin KO HT1080 cells were restimulated after the starvation for 3 hours with glucose containing medium or starved for 12 hours for glucose in the presence of Dorsomorphin. Cells were stained with LAMP1 (green) and EEA1 (red). Scale bars: 10 μm. (B) Quantification of the percentage of cells that display HT1080 characteristic enlarged late endosomes in steady state or upon 12 hours glucose starvation. Cells were counted from at least three biological replicates with over 100 cells per genotype and tested condition pro replica. Data presented as mean values ± SD. Unpaired Student's *t* test was performed between each condition in each genotype (*P ≤ .05; **P ≤ .01; ***P ≤ .001). (C) Quantification of the percentage of cells that display HT1080 characteristic enlarged late endosomes in steady state, upon 12 hours glucose starvation, 12 hours glucose starvation followed by 3 hours glucose restimulation and upon 12 hours glucose starvation in the presence of Dorsomorphin. Cells were counted from at least three biological replicates with over 100 cells per genotype and tested condition pro replica. Data presented as mean values ± SD. Unpaired Student's *t* test was performed between each condition in each genotype (*P ≤ .05; **P ≤ .01; ***P ≤ .001). (D) Representative WB of WT and Diaskedin KO HT1080 cells either in steady state, upon 12 hours glucose starvation and 12 hours glucose starvation followed by 3 hours glucose restimulation

Therefore, we next tested if the activation of AMPK by glucose starvation would also affect endosomal size. Therefore, we starved HT1080 WT, Diaskedin KO, Myrlysin KO and Lyspersin KO cells for glucose^19^, ^30^ and analyzed the percentage of cells with enlarged endosomes (Figure 8A,B). Much like the Diaskedin deletion mutant, which has intrinsically higher levels of AMPK signaling (Supplementary Figure S4A) and smaller late endosomes/lysosomes (Figure 2), glucose‐starved WT HT1080 now also reduced enlarged late endosomes, compared to nonstarved controls. This phenotype was quickly reverted when cells were placed back in high glucose containing medium (Figure 8A,C). Glucose starvation caused an increase in pAMPK in WT HT1080 cells, which was exacerbated upon Diaskedin deletion (Figure 8D). Upon glucose restimulation, pAMPK levels decreased again (Figure 8D). When cells were treated with the AMPK inhibitor Dorsomorphin in combination with glucose starvation, there was a prominent increase of the number of cells that displayed enlarged late endosomes (Figure 8A,C). The increase in cells, bearing characteristic enlarged late endosomes, upon AMPK inhibition by Dorsomorphin was also observed in HT1080 WT and Diaskedin KO cells under full growth conditions (Supplementary Figure S4B,C). Here, we treated cells with the chemical inhibitors Dorsomorphin and PD0325901, which either block AMPK or MAPK signaling, respectively, and scored for the percentage of cells that displayed enlarged late endosomes. When incubating WT and Diaskedin KO cells with Dorsomorphin, we noticed a significant increase of the number of cells with enlarged late endosomes in both genotypes, compared to steady state cells (Supplementary Figure S4B,C). On the contrary, upon using the MEK inhibitor PD0325901 we saw the opposite, with clearly less cells showing enlarged endosomes, particularly in the WT genotype (Supplementary Figure S4B,C). This reciprocal effect of the two inhibitors used, could be explained by the fact that AMPK and MAPK signaling have been demonstrated to have a reciprocal negative effect on each other.^39^ Indeed, when total cell lysates of Dorsomorphin‐ or PD0325901‐treated cells were analyzed by Western blotting for pERK or pACC, as direct targets for either MAPK or AMPK activity, respectively, this effect could be recapitulated (Supplementary Figure S4D). Cells, treated with Dorsomorphin had decreased activity of AMPK compared with cells in steady state, but displayed hyperactivated MAPK signaling and vice versa: cells treated with PD0325901 had an almost complete block of MAPK signaling, but hyperactivated AMPK. Importantly, Diaskedin KO HT1080 cells showed consistent increase of pACC, further supporting our observation that these cells have intrinsically increased AMPK signaling. Taken together, glucose starvation as a trigger for AMPK activation reduced late endosomal/lysosomal size, possibly by controlling PIKfyve dependent PI(3,5)P_2_ levels and lysosomal reformation.

### 2.9 Enhanced lysosomal reformation correlates with autophagy induction in BORC deficient cells

**Figure 9:**
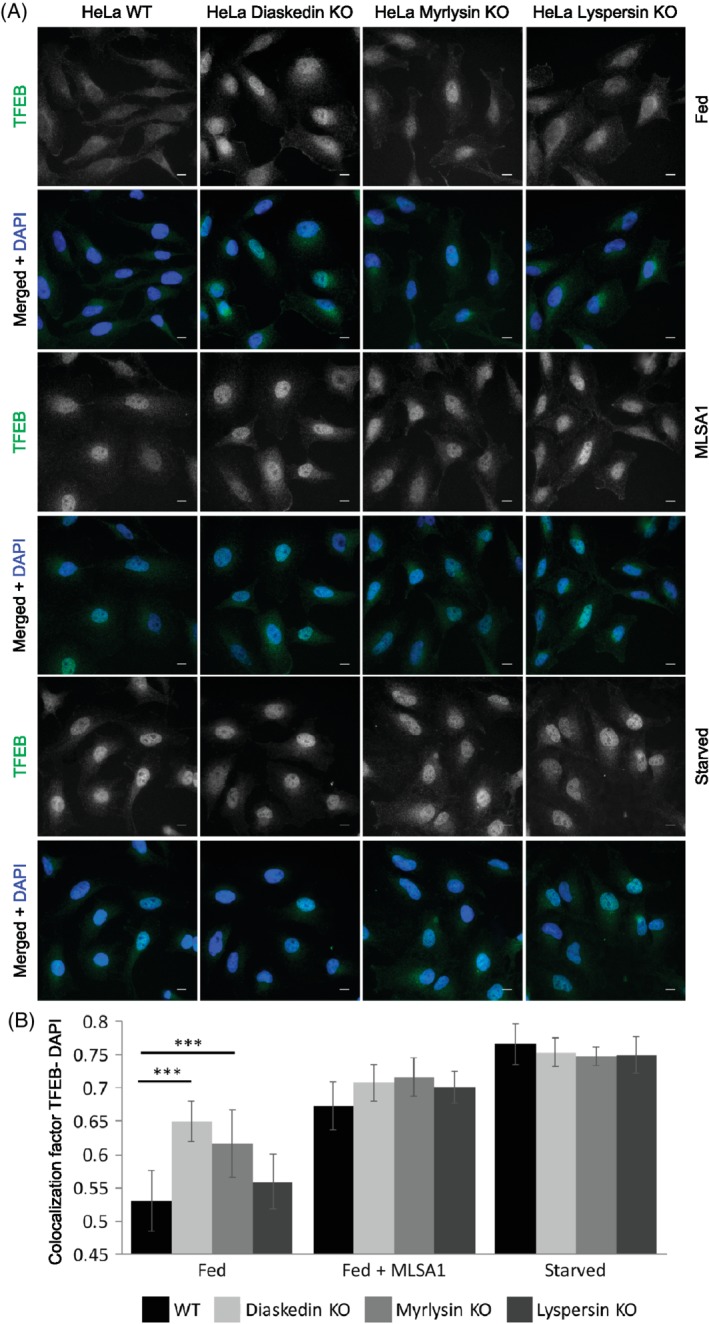
Increased nuclear TFEB translocation upon Diaskedin and Myrlysin but not Lyspersin deletion in HeLa cells. (A) IF of WT, Diaskedin KO, Myrlysin KO and Lyspersin KO HeLa cells, stained for endogenous TFEB (green) and the nuclear marker DAPI (blue) either under normal feeding (fed) condition, under fed condition in the addition of 10 μM ML‐SA1 or upon 3 hours AA and FBS starvation. Scale bars: 10um. (B) Quantification of the TFEB‐DAPI colocalization, based on at least 10 frames, acquired from two biological replicates in each corresponding genotype and condition. Data presented as mean values ± SD. Unpaired Student's *t* test was performed between each genotype pro condition (*P ≤ .05; **P ≤ .01; ***P ≤ .001)

**Figure 10:**
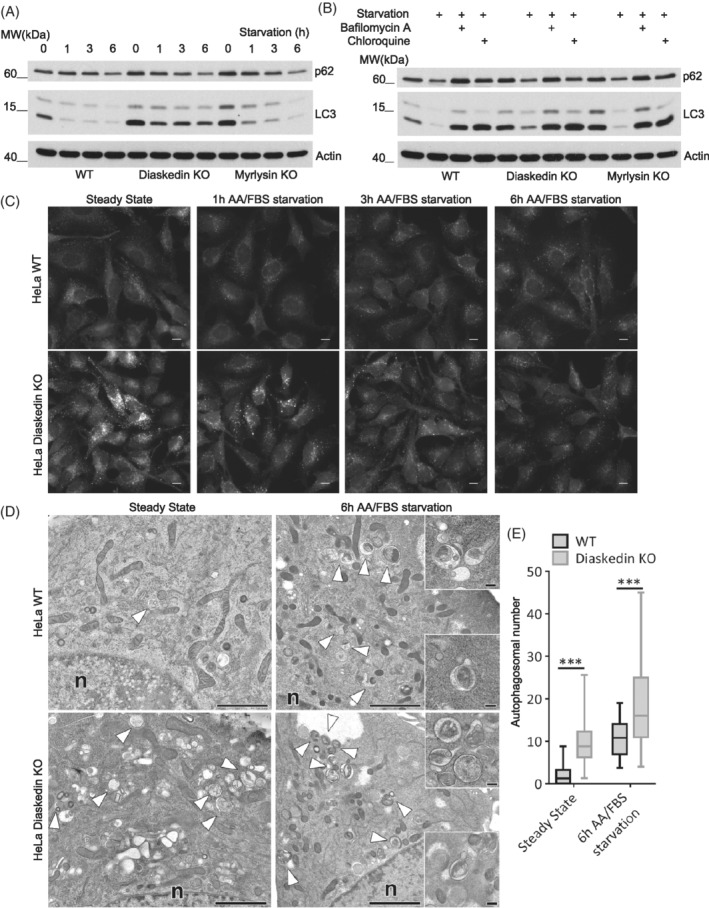
Increased baseline autophagy upon Diaskedin and Myrlysin deletion in HeLa cells. (A) WB analysis of WT, Diaskedin KO and Myrlysin KO HeLa cells, either under steady state or upon AA/FBS starvation for the indicated time points (h). (B) WB analysis of WT, Diaskedin KO and Myrlysin KO HeLa cells, either under steady state, upon 6 hours AA/FBS starvation or upon 6 hours AA/FBS starvation in the presence of the indicated chemical inhibitors. (C) IF of WT and Diaskedin KO HeLa cells, stained for endogenous autophagic marker LC3 either under steady state or upon the indicated time of AA/FBS starvation. Scale bars: 10 μm. (D) EM of cryofixed HeLa WT and Diaskedin KO cells at steady state, as compared to 6 hours AA/FBS starvation. In WT cells autophagosomes (white arrow‐heads) are very rare at steady state, but increase in number after starvation. However, Diaskedin KO cells contain numerous autophagosomes already at steady state, and even more after 6 hours AA/FBS starvation. Inserts show autophagosomes with distinct buds and tubes considered to characterize lysosomal reformation^59^ as a consequence of starvation. Scale bars: 2 μm in overviews, 200 nm in inserts. (E) Frequency of autophagosomes per 100 square‐micron cytoplasm in WT and Diaskedin KO HeLa as counted on EM images. Autophagosomes from over 25 cells per genotype were counted. Data presented as median value with the box representing the 25th and 75th percentile, and the whiskers—the minimal and maximal values. Unpaired Student's *t* test was performed between each condition in each genotype (*P ≤ .05; **P ≤ .01; ***P ≤ .001)

Having established that lysosomal biogenesis was severely altered upon deletion of core BORC components, we next investigated whether this would also affect autophagy, since lysosomal reformation and autophagy are tightly interconnected and depend on each other.^40^ The lysosomal calcium channel Mucolipin‐1 (ML‐1), also known as TRPML1 (transient receptor potential cation channel, Mucolipin subfamily, member 1), has been shown to be required for proper TFEB dephosphorylation and thus TFEB translocation into the nucleus, which consequently is a prerequisite for autophagy initiation. Interestingly, ML‐1 activation is dependent on PI(3,5)P_2_ levels on lysosomes.^41^, ^42^ This led us to the hypothesis that increased PI(3,5)P_2_ production, observed in Diaskedin and Myrlysin deletion mutants and the resulting higher ML‐1 activation, would translate into more dephosphorylation and thus activation with subsequent nuclear accumulation of TFEB. Indeed, under normal feeding conditions, Diaskedin and Myrlysin depleted HeLa cells displayed significantly higher nuclear TFEB localization, compared to WT and Lyspersin KO cells (Figure 9A,B) and this effect could be mimicked by treating cells with a synthetic Mucolipin‐1 activator (ML‐SA1).^43^ Under starvation conditions all four genotypes displayed similar high nuclear TFEB localization. The stronger nuclear TFEB signal in Diaskedin and Myrlysin deletion mutants (Figure 9A,B) would be suggestive of increased baseline autophagy. To test this assumption, we analyzed the lipidation status of the autophagy marker LC3 (microtubule‐associated proteins 1A/1B light chain 3B).^44^ Under steady state, Diaskedin and Myrlysin deletion mutants had higher levels of lipidated LC3 compared to WT, indicative of active autophagy (Figure 10A,C). Over a 6 hours AA and fetal bovine serum (FBS) starvation time course the levels of lipidated LC3 were reduced, presumably because of LC3 consumption in lysosomes (Figure 10A,C). When the lysosomal inhibitors Bafilomycin A or CQ were added in combination with 6 hours AA and FBS starvation, lipidated LC3 became less efficiently degraded and accumulated to comparable amounts in all genotypes (Figure 10B). EM analysis after high pressure freezing of WT and Diaskedin KO HeLa cells under steady state and upon 6 hours AA and FBS starvation allowed us to visualize and count autophagosomes in these cells (Figure 10D,E). Importantly, Diaskedin KO cells had consistently more autophagosomes already at steady state and those numbers increased further during starvation, corroborating our hypothesis that these cells display increased baseline autophagy.

We concluded that deletion of the two BORC subunits Diaskedin and Myrlysin resulted in hyperaction of AMPK, which in turn activated PIKfyve. Higher levels of PI(3,5)P_2_ enforced ML‐1 dependent transcription factor (TFEB) activation, initiated autophagy and enhanced lysosomal reformation. As these processes are no longer restrained by BORC, this ultimately led to shrinking and increasing number of lysosomes.

## 3 DISCUSSION

### 3.1 BORC regulates late endosomal/lysosomal size via PI(3,5)P_2_

The multimeric BORC localizes to the limiting membrane of late endosomes/lysosomes and controls organelle positioning, through the recruitment of the small Arf‐like GTPase Arl8b^2^. All components of the BORC complex investigated (Diaskedin, Myrlysin and Lyspersin), contribute to the same extent to endosomal positioning through Arl8b recruitment (Figure 3C).^14^, ^15^ In the present contribution, we have shown that two BORC subunits, Diaskedin and Myrlysin exerted an additional effect on endosomal size via PI(3,5)P_2_. Cells, bearing those specific BORC deletions have elevated PI(3,5)P_2_ levels and in consequence contained more and smaller late endosomes/lysosomes. Interestingly, this phenotype was not equally shared by Lyspersin deletion mutants. Based on our results, we propose that the BORC components Diaskedin and Myrlysin fulfill a so far unexplored function in endosomal size regulation, in addition to their contribution to Arl8b recruitment and endosomal positioning.

In particular, we found that members of the BORC complex negatively regulate PIKfyve activity and thereby affect PI(3,5)P_2_ levels and consequently late endosomal/lysosomal size. The direct quantification of PtdInsP species revealed that PI(3,5)P_2_ was highly enriched in Diaskedin KO cells (Figure 4C and Supplementary Figure S3D). This could be caused either by activating the lipid kinase PIKfyve or by inhibiting the counteracting phosphatase Sac3. As both the kinase and the phosphatase reside within the same conserved protein PIKfyve‐ArPIKfyve‐Sac3 (PAS) complex,^45^, ^46^, ^47^ future studies should resolve in detail how exactly BORC interfaces with the PIKfyve containing PAS complex to regulate PI(3,5)P_2_ production. Based on our results, we propose a model in which enhanced PI(3,5)P_2_ production leads to more active lysosomal reformation and/or fission, which has recently been linked to PIKfyve activity.^17^, ^18^ Therefore, cells bearing a Diaskedin deletion, undergo too frequently lysosomal fission and reformation. Therefore, these cells have (a) smaller and (b) more late endosomes.

Furthermore, our results suggest that AMPK functions as a possible activator of PIKfyve in PI(3,5)P_2_ synthesis, driving endosomal reformation and thus leading to reduction of endosomal size. This conclusion is based on several independent observations. First, AMPK KO in HT1080 cells led to increased numbers of characteristic enlarged endosomes (Figure 6A‐C). Concomitantly, Dorsomorphin‐mediated inhibition of AMPK led to reduced PI(3,5)P_2_ production (Figure 6D) and simultaneously to an increase in the frequency of enlarged endosomes in HT1080 cells (Figure 8A,C and Supplementary Figure S4B,C). Conversely, AMPK activation by means of glucose starvation had the opposite effect and characteristic, enlarged late endosomes of HT1080 cells were dramatically reduced (Figure 8). As AMPK has been shown to directly phosphorylate the sole PI(3,5)P_2_ producing kinase PIKfyve,^32^ we envision that the observed increase in PI(3,5)P_2_ in Diaskedin KO HT1080 (Figure 4C and Supplementary Figure S3D) is caused by the intrinsic increase of AMPK activity in these cells (Supplementary Figure S4A,D). In light of these findings, we tested whether altered PI(3,5)P_2_ levels would vice versa have an effect on the BORC complex. For this we incubated WT HeLa cells with the previously described PI(3,5)P_2_ inhibitors VPS34IN and YM201636 (Supplementary Figure S3A) and performed subcellular fractionation to detect possible changes in BORC expression and localization. Based on the observation that inhibition of PI(3,5)P_2_ by those inhibitors led to the decrease of BORC components specifically at membrane fractions (Supplementary Figure S5A,B), it is tempting to speculate that PI(3,5)P_2_ levels could regulate BORC stability and/or localization in a feedback mechanism and thereby ultimately control lysosomal reformation and late endosomal/lysosomal size reduction.

### 3.2 Glucose levels regulate late endosomal/lysosomal size

When subjected to energy stress, cells react by reprogramming anabolic and catabolic processes, favoring the latter to maintain cellular homeostasis and to survive stress conditions. The endolysosomal system is a main regulator of those processes with a central role for the lysosomal residential LAMTOR/Ragulator complex in sensing intracellular AA and glucose levels and subsequent lysosomal mTORC1 activation.^28^, ^29^, ^48^ Based on our work, we propose that components of the BORC complex fulfill additional functions, which are linked to LAMTOR/Ragulator. Previously we identified a LAMTOR/Ragulator deletion phenotype, which is manifested by an increased number and also decreased size of late endosomes/lysosomes.^36^ Here, we describe that LAMTOR/Ragulator interacting BORC components could be responsible for the observed phenotype. In our epistasis experiments (Figure 7), where late endosomal/lysosomal size was taken as a readout, LAMTOR/Ragulator deletion highly phenocopied BORC deletion, albeit to a lesser extent. Importantly, when the BORC core component Diaskedin was deleted in LAMTOR2 deficient cells (double KO), there was a further decrease of late endosomal/lysosomal size. This could indicate that primarily components of the BORC complex are responsible for the regulation of late endosomal/lysosomal size and LAMTOR/Ragulator fulfills modulating functions. Alternatively, since LAMTOR and BORC display a synthetic/synergistic phenotype on late endosomal/lysosomal size, this could indicate that BORC and LAMTOR function in two parallel pathways that control late endosomal/lysosomal size independently. In contrast, for the distribution of late endosomes, it is clear that BORC is upstream of LAMTOR and that both complexes work together to position late endosome/lysosomes. Deletion of LAMTOR/Ragulator is associated with translocation of late endosomes/lysosomes toward the cell periphery.^27^ Notably, this distinct mislocalization phenotype was dependent on BORC (Figure 7B).

In our current work, we demonstrated that energy stress, triggered by glucose deprivation, is accompanied by significant morphological changes of the endolysosomal system, in particular size reduction of late endosomes/lysosomes. This is most likely achieved by inducing lysosomal reformation via PIKfyve activation and subsequent PI(3,5)P_2_ production and could have several beneficial effects for the cell. For instance, autophagy, a process which is also boosted during glucose starvation, requires newly formed lysosomes to efficiently degrade cargo molecules and thereby recycle metabolic building blocks on which cells rely during nutrient shortage. Surprisingly, the acute morphological changes in the endolysosomal system following glucose limitation were reminiscent of Diaskedin deletion (Figure 8A,B). Accordingly, late endosomes/lysosomes of Diaskedin KO cells appeared to be constantly undergoing lysosomal reformation, even when not under starvation stress. Therefore, we propose that the BORC might participate in an energy sensing machinery, which ultimately affects endosomal size and morphology.

Recently Zong and colleagues suggested that glucose sensing by the lysosomal AMPK activation pathway serves as a surveillance system monitoring the availability of glucose and switching off anabolic pathways.^38^ Our data might provide a link for the lysosomal noncanonical glucose‐sensing role of AMPK^30^ toward the executing BORC machinery for late endosomal/lysosomal adaption. We propose a model (Supplementary Figure S5C) in which low energy levels, caused for instance by glucose starvation, activate lysosomal reformation via the “BORC‐AMPK‐PIKfyve‐ML‐1 axis.” BORC is embedded in regulatory circuitry involving AMPK, PIKfyve, ML‐1, TFEB and autophagy. Activating one of those players could potentially set in motion an entire feed‐forward feedback mechanism. Upon glucose restimulation, BORC is then required to limit lysosomal reformation by negatively regulating AMPK‐dependent PIKfyve activation. In Diaskedin deletion mutants, this process is no longer restricted and might lead to a constant energy stress‐like status, which is manifested by activated AMPK signaling and subsequently increased PIKfyve activity and lysosomal reformation. Furthermore, constant energy stress‐like status also leads to increased baseline autophagy, which we observed in Diaskedin KO and Myrlysin KO cell lines. While TFEB dephosphorylation and its subsequent nuclear translocation can promote autophagy, it also promotes lysosomal biogenesis including regulation of LAMP1 levels, which might explain why some BORC deletion mutants display higher LAMP1 levels (Figure 1A, 2A and Supplementary Figure S1D). This is manifested by increased autophagosome generation and we believe that it is functionally linked to tubule formation and the resulting reduction of endosomal size (Supplementary Figure S5C). Both endosomal/lysosomal size regulation and tubule formation as well as baseline autophagy are themselves dependent on PI(3,5)P_2_ production, which we demonstrated to be controlled by the activity of BORC, the LAMTOR/Ragulator and AMPK complexes. Defects in PI(3,5)P_2_ synthesis have been linked to a variety of neurodegenerative diseases, including amyotrophic lateral sclerosis (ALS) and Charcot‐Marie‐Tooth (CMT) disease.^49^, ^50^, ^51^ Lysosome reformation may occur in one of two processes: (a) as part of the baseline lysosome‐endosome fusion‐fission cycles and (b) reformation from spent autolysosomes as in ALS. Therefore, one could envision the findings presented here as a basis for exploring possibilities to treat such conditions.

Note added in proof: Recent work by others provided complementary insight into the impact of PIKfyve on endolysosomal homeostasis (Sharma G, Guardia CM, Roy A, et al. A family of PIKFYVE inhibitors with therapeutic potential against autophagy‐dependent cancer cells disrupt multiple events in lysosome homeostasis. Autophagy. 2019:1‐25.).

## 4 MATERIALS AND METHODS

### 4.1 Reagents

Primary antibodies were obtained from the following sources and used according to the manufacturers' instructions: rabbit anti‐Diaskedin (WB 1:1000; HPA037648; SIGMA), rabbit anti‐Myrlysin (WB 1:1000; AP5806B‐AB; BioCat), rabbit anti‐Lyspersin (WB 1:1000; HPA045415, SIGMA), rabbit anti‐Snapin (WB 1:1000; 10 055‐1‐AP, Proteintech), mouse anti‐LAMP1 (WB 1:2000; 555 801, BD Pharmingen), mouse anti‐LAMP1 (IF 1:250; H4A3, Developmental Studies Hybridoma Bank), rabbit anti‐EEA1 (IF 1:250; sc‐33 585, Santa Cruz), mouse anti‐CD63(IF 1:250; H5C6, Developmental Studies Hybridoma Bank), mouse anti‐M6PR (IF 1:250; 22D4, Developmental Studies Hybridoma Bank), mouse antitubulin (WB 1:2000; 12G10, Developmental Studies Hybridoma Bank), mouse antiactin (WB 1:5000; MAB1501, Merck Millipore), mouse anti‐TfR (WB 1:1000; G1/221/12, Developmental Studies Hybridoma Bank), mouse anti‐pERK1/2(T202/Y204) (WB 1:1000; 9101, Cell Signaling), rabbit anti‐ERK1/2 (WB 1:1000; 9102, Cell Signaling), rabbit anti‐p70S6K(T389) (WB 1:1000; 9234, Cell Signaling), rabbit anti‐p70S6K (WB 1:200; sc‐230, Santa Cruz), rabbit anti‐pS6(S240/244) (WB 1:2000; 2215, Cell Signaling), rabbit anti‐pAMPK(T172) (WB 1:1000; 2535, Cell Signaling), rabbit anti‐AMPK (WB 1:1000; 2603, Cell Signaling), rabbit anti‐pACC(S79) (WB 1:1000; 3661, Cell Signaling), rabbit anti‐ACC (WB 1:1000; 3676, Cell Signaling), rabbit anti‐TFEB (IF 1:250; 4240, Cell Signaling), rabbit anti‐LC3 (WB 1:1000; 2775, Cell Signaling), rabbit anti‐LC3 (IF 1:1000; PM036, MBL) and rabbit anti‐p62(SQSTM1) (WB 1:1000; 5114, Cell Signaling). For Western blotting, secondary antibodies horseradish peroxidase (HRP)‐conjugated goat antimouse (SIGMA A4416, 1:10 000) and HRP‐conjugated goat antirabbit (SIGMA A0545, 1:10000) were used. Alexa Fluor 488 conjugated goat antimouse (Thermo Fischer Scientific #A‐11001), Alexa Fluor 568 conjugated goat antirabbit (Thermo Fischer Scientific #A‐11036) and Oregon Green 488 conjugated goat antimouse (Thermo Fischer Scientific #O‐11033) at a dilution 1:1000 were used for immunofluorescence (IF). Additional reagents were obtained from the following sources and used at the indicated concentration: Chloroquine (C6628, SIGMA, 50 μM), bafilomycin A1(B1793, SIGMA, 40 nM), rapamycin (553 210, Merck, 100 nM), PMA (AG‐CN2‐0010, BioMol, 1 μg/mL), DQBSA (D12051, Invitrogen, 10 μg/mL), VPS34IN (Cay‐17 392, BioMol, 2.5 μM), YM201636 (inh‐ym20, InvivoGen, 800 nM), Dorsomorphin (P5499, SIGMA, 100 μM), PD0325901 (S1036, Absource, 100 nM) and AICAR (A9978, SIGMA, 1 mM).

### 4.2 Generation of CRISPR/Cas9 knockout cell lines

The pLentiCRISPRv2‐LoxPv1 plasmid was generated by inserting LoxP sites (5′ATAACTTCGTATAGCATACATTATACGAAGTTAT‐3′) flanking the elongation factor 1α short (EFS) promotor of the pLentiCRISPRv2 plasmid (Addgene 5261).^52^ Guide RNA (gRNA) sequences targeting *Homo sapiens Diaskedin* (5′‐GGTTCGGTCAGTCCGTGAAG‐3′), (*myrlysin*) (5′‐CCGATATCAAGATCACCTGC‐3′), *lyspersin*(5′‐GCGTCGTCCACCGGCCGGAA‐3′), *PRKAA1*(5′‐CCGAGAAGCAGAAACACGA‐3′) and *Mus musculus Diaskedin*(5′‐GATTTGGTCAGTCCGTGAAG‐3′) were selected, based on the online gRNA prediction tool (http://crispr.mit.edu/).^53^ The gRNAs were subsequently subcloned in pLentiCRISPRv2‐LoxPv1 and used for lentiviral production. For production of lentivirus containing the CRISPR/Cas9 and the selected gRNAs, HEK293LTV cells were used. These were cotransfected with pLentiCRISPRv2‐LoxPv1, containing a single gRNA together with pVSV‐G (Clonetech 631 530) and psPAX2. The supernatant was used to directly infect target cells, which were subsequently selected with 1ug/mL puromycin (Sigma P7255). HT1080 Lyspersin KOs, HT1080 Myrlysin KOs, HT1080 LAMTOR2 KOs and HT1080 Double KOs (Diaskedin and LAMTOR2) were used as “bulk” KO. For the generation of the HT1080 Diaskedin KOs and the HeLa Diaskedin KOs, the cells were further transiently transfected with pCAG‐Cre‐GFP (Addgene 13 776) to remove the Cas9 and fluorescence‐activated cell sorting (FACS) sorted to enrich for the GFP positive population, expressing high Cre amounts. These were then allowed to recover for 24‐48 hours before they were seeded at a concentration below 5cells/mL in 200 μL in 96well plates. The single cell clones were tested by a polymerase chain reaction (PCR) screen^54^ and by Western blotting for the loss of the target gene.

### 4.3 Plasmid generation

The template for generating a rescue HA‐Diaskedin construct was generated by reverse transcription and complementary deoxyribonucleic acid synthesis from messanger ribonucleic acid extracts from HT1080WT cells. Two specific digestion sites (NotI and XbaI) were introduced, flanking the open reading frame (ORF) of Diaskedin. These were then used to insert Diaskedin ORF into pENTR4 (Invitrogen A10465), bearing an N‐terminal HA‐tag. The construct was recombined, using a GATEWAY LR reaction (Invitrogen), into pCCL‐EFS‐Blasti‐DEST mammalian expression vector. Similarly, a C‐terminally tagged version of Myrlysin was generated, using specific digestion sites (NcoI and BamHI) on pENTR4 (Invitrogen A10465) vector, bearing a C‐terminal Ruby tag. This was then recombined, using a GATEWAY LR reaction (Invitrogen), into pCCL‐EF1Alpha‐DEST mammalian expression vector. A full length Arl8b was cloned into pENTR207 (Invitrogen 12213‐013) vector, excluding the stop codon. This was then shuttled to pEGFP‐N‐DEST vector using GATEWAY technology (Invitrogen). pEGFP‐N‐DEST was itself generated from pEGFP‐C1 (Clontech), modified by inserting a GATEWAY destination cassette (Invitrogen) into the NheI site upstream of the enhanced green fluorescent protein coding sequence. The generation of LAMP1‐Neon Green was performed as follows: full‐length rat LAMP1—fused to mNEONGreen (Allele Biotech, San Diego, USA) was amplified by PCR, subcloned into BamH1‐NotI sites of pENTR4 (Invitrogen A10465) and then shuttled to pQXCIN‐DEST using GATEWAY technology (InVitrogen). pQXCIN itself was generated from pQCXIN (Clontech), modified into a GATEWAY compatible destination vector by inserting a GATEWAY destination cassette into the AgeI site upstream of the IRES‐NEO cassette. G glycoprotein of vesicular stomatitis virus (VSV‐G) pseudotyped retroviral virions were generated by transient transfection of amphotropic Phoenix packaging cells^55^ and used to infect target cells. Transduced cells were selected against 1 mg/mL G418.

### 4.4 Subcellular fractionation

Cells grown on 15 cm petri dishes were cooled on metal plates on ice and washed with ice cold phosphate buffer saline (PBS). PBS, containing 0.5x protease inhibitors was added and cells were scraped, transferred to Falcon tubes and centrifuged in a precooled centrifuge at 160*g* for 5 minutes. The supernatant was removed and the pellet wash washed (without disturbing its integrity) with Homogenization Buffer (250 mM sucrose and 3 mM imidazole in H_2_O), supplemented with 1 mM ethylenediaminetetraacetic acid (EDTA), 30ug/mL cycloheximide and 1x protease inhibitors (HB+ buffer). Upon another centrifugation step at 690*g* for 10 minutes, the supernatant was removed and cells were completely resuspended in HB+ buffer, using three times the volume of the pellet. Cells were then homogenized using a 25‐G needle, attached to a 1 mL syringe. Nuclei were pelleted at 1000*g* for 10 minutes. The postnuclei supernatant (PNS) was further centrifuged at 100000*g*; the supernatant was collected as cytosolic fraction and the pellet was resuspended in equal volume homogenization buffer and labeled as total membranes. For the isolation of crude endosomal (CE) fraction, PNS was loaded on the bottom of SW41 centrifuge tubes (Beckman Coulter). The sucrose concentration of the PNS fraction was adjusted to 40.6% by adding about 1.2x the volume of the PNS of 62% sucrose. The mixture was overlaid with 7‐8 mL of 35% sucrose (supplemented with protease inhibitors and cycloheximide). The last few millimeters of the tube were filled with homogenization buffer, supplemented with protease inhibitors. The samples were centrifuged for 3 hours at 34 000 rpm at 4°C in a Beckman Coulter ultracentrifuge, using SW41 rotor. Upon centrifugation, 750 μL of the upper fraction was collected, 500ul of 3 mM Imidazole (pH 7.4) was added and centrifuged again for 30 minutes at 100 000*g* at 4°C. The supernatant was discarded and the pellet was resuspended in 30ul homogenization buffer containing protease inhibitors and labeled as CEs.

### 4.5 Cell culture

Cells were cultured in Dulbecco's modified Eagle's medium (DMEM) with high glucose (SIGMA D6429) or alternatively for glucose starvation in DMEM without glucose (Thermo Fischer Scientific 11966025), supplemented with 10% FBS (Gibco 10 270) and 100 U/mL penicillin and 100 mg/mL streptomycin (SIGMA P0781) at 37°C, in 5% CO_2_ and 95% humidity. For trypsination of the cells, a Trypsin‐ EDTA solution (SIGMA T4174) and homemade PBS was used. Stable cell lines, expressing HA‐Diaskedin (Rescue) were supplemented with 10 μg/mL blasticidin and “bulk” KO cell lines were selected in media containing 1 μg/mL Puromycin.

### 4.6 Immunofluorescence and live cell microscopy

Cells, grown on glass cover slips, were fixed in 4% formaldehyde solution in PBS for 10 minutes and subsequently washed with PBS. The cells were then permeabilized in blocking buffer (0.05% saponin in PBS with 10% goat serum [SIGMA G6767]) for 1 hour at room temperature (RT); incubated for 1 hour at RT with primary antibodies, diluted in blocking buffer; washed several times with PBS; incubated for further 1 hour at RT with secondary antibodies, diluted in blocking buffer; washed once briefly with PBS containing 4′,6‐diamidino‐2‐phenylindole; washed several more times with PBS and mounted on microscope slides using Mowiol. Alternatively, cells were permeabilized in 0.2% Triton in PBS for 5 minutes and no detergents were used in the subsequent steps. Still images were acquired on the Axio‐Imager M1 microscope (Carl Zeiss) with a 100x oil immersion objective (NA 1.45), equipped with a SPOT Xplorer, Visitron Systems camera at RT and movies on an inverted Zeiss Axiovert 200 M microscope with a 63x oil immersion objective (NA 1.4), equipped with Cool SNAP HQ_2_ Photonics interline CCD camera in a 37°C heated chamber using Leibovitz's phenol free medium (Invitrogen 21083‐027). Acquisition was controlled by VisiView software (Visitron Systems GmbH). Single plane confocal images and Z stacks were acquired using a Laser Scanning Confocal Microscope (SP5, Leica) with a 63x oil immersion objective (NA 1.4) at RT. For super resolution imaging a gated STED with a pulsed white light laser (SP8 gSTED, Leica) microscope with a 63x oil immersion objective (NA 1.4) was used. Still images were acquired at RT and movies in a 37°C heated chamber using Leibovitz's phenol free medium (Invitrogen 21083‐027). Still images and movies, acquired at the SP8 gSTED, were deconvoluted with Huygens Professional Deconvolution and Analysis Software (Scientific Volume Imaging). All images were reformatted to TIFF (respectively time series to movies) using ImageJ; brightness and contrast on all images were adjusted using Adobe Photoshop CS5.

### 4.7 EM, tomography, immunogold labeling and ultrastructural morphometry

Specimen preparation, immunoelectron microscopy and electron tomography were performed as previously described in detail.^36^, ^56^ Briefly, for morphology, the cells were cultured on sapphire discs suitable for high‐pressure freezing,^57^ freeze substitution and epoxy resin embedding. Thin sections (80 nm) were used for standard TEM, and 300‐400 nm‐semi‐thick sections for dual‐tilt scanning transmission electron tomography (STEM, with a TECNAI T20G2 from FEI, now: Thermo Scientific, Waltham, MA, USA). For immunogold EM, we used Tokuyasu‐cryosection labeling^58^ or our recently described method for cryobased pre‐embedding immunogold labeling.^56^ Primary antibodies were mouse antihuman LAMP1 (EM: 1:10; H4A3; Developmental Studies Hybridoma Bank), rabbit anticathepsin D (EM: 1:100; Ab‐2; Calbiochem), mouse anti‐HA (1:30‐1:100; MMS‐101R; Covance), secondary antibodies were NANOGOLD‐Fab’ goat antimouse and NANOGOLD‐Fab' goat antirabbit IgG (H + L) (1:150; #2002, 2004, respectively, both from Nanoprobes), goat antimouse IgG 5 nm gold (#EM.GAM5, British Biocell) and visualized by silver enhancement (SE) with HQ‐Silver (#2012, Nanoprobes). Processing of digital electron micrographs was optionally carried out with iTEM‐analySIS five software (from EMSIS, Münster, Germany) or Adobe Photoshop V.9, including adjustment of contrast and brightness, gray‐scale modification, sharpening, median and high‐pass filtering. For morphometry, we used samples from >2 independent cell culture experiments (ie, n = 2, with several technical replicates each). Morphometry was carried out on digital micrographs with a MORADA camera (from OSIS) taken at primary magnifications of x11 500 by using measurement tools of the iTEM software.

### 4.8 Phosphoinositide labeling and HPLC‐coupled flow scintillation

HT1808 WT, HT1080 Diaskedin, Myrlysin and Lysperin KO cells were incubated for 24 hours in tritium labeling medium (inositol‐free DMEM [MP Biomedical]) supplemented with 15 μCi/mL *myo*‐(2‐^3^H[N]) inositol (Perkin Elmer), 4 mM L‐glutamine (Sigma‐Aldrich), 1x insulin‐transferrin‐selenium‐ethanolamine (Gibco), 10% dialyzed FBS (Gibco) and 1x penicillin‐streptomycin mix (Sigma‐Aldrich). After, labeling medium was removed and replaced with cell culture medium, where cells were incubated in the presence or absence of 200 nM Apilimod (Toronto Research Chemicals) for 1 hour or 10 μM Dorsomorphin for 3 hours. Following, cells were lysed on ice with 4.5% perchloric acid (vol/vol) for 15 minutes then pelleted at 12 000*g* for 10 minutes at 4°C. Pellets were washed with 1 mL of ice‐cold 100 mM EDTA, pH 8.0, then sonicated in 50 μL of water. Samples were incubated in phospholipid deacylation reagent (10.7% methylamine, 45.7% methanol and 11.4% 1‐butanol [vol/vol]) for 60 minutes at 53°C. Samples were washed in water twice and dried with a speed vacuum. Dried samples were resuspended in a 1.5:1 ratio of water to extraction reagent (1‐butanol/ethyl ether/ethyl formate [20:4:1]), vortexed for 5 minutes, and centrifuged for 2 minutes at max speed. The aqueous layer was isolated and extracted twice more, then vacuum dried and resuspended in 50 μL of water. Samples were then separated by HPLC (Agilent Technologies). For each sample, equal counts of tritium were separated using an anion exchange 4.6 × 250‐mm column (Phenomenex) and subjected to a gradient of 1 M (NH_4_)_2_HPO_4_, pH 3.8 and water. For details of HPLC separation protocol see Ho et al. (2016).^60^ The radiolabeled eluate was detected by β‐RAM 4 (LabLogic) at a 2:1 ratio of scintillation fluid to eluate and analyzed with Laura 4 software. For quantification, all phosphoinositide species levels were normalized against the parent phosphatidylinositol peak.

### 4.9 Quantification and statistical tests

Endosomal count in HeLa cells was based on 10 cells per phenotype, where endosomes were automatically counted using Imaris software. Endosomal diameter in HeLa cells was based on measurements of at least 100 endosomes from at least six cells per genotype imaged. The percentage of cells, displaying enlarged late endosomes in HT1080 cells was determined, based on acquired immunofluorescent images, using LAMP1 antibody, where at least 300 cells from a minimum of three biological replicates were assigned to either “normal” or “enlarged” group based on general size and abundancy of late endosomes. These were quantified by applying an unpaired Student's *t* test (*P ≤ .05; **P ≤ .01; ***P ≤ .001).

## AUTHOR CONTRIBUTIONS

T.E.Y. as first author designed and performed experiments, and wrote the manuscript. V.E.B.H., M.W.H., G.F.V. and C.H. designed and performed experiments. S.G. designed and provided constructs, G.L. contributed to the statistical analyses. T.S., D.T. and R.J.B. designed experiments, contributed conceptually and in the writing of the manuscript. L.A.H. initiated and supervised the project, designed experiments and wrote the manuscript together with M.W.H. as corresponding authors.

## Supporting information


**Figure S1.** An overview of the endosomal system of HT1080 WT cells, labeling of oversized endocytic compartments in HT1080 WT cells with lysosomal markers and a subcellular fractionation of BORC deletion mutants.
**Figure S2**. Effect of Chloroquine and a selection of other chemical inhibitors on the frequency of the characteristic enlarged late endosomes in BORC deletion mutants in HT1080.
**Figure S3.** Schematically where VPS34IN and YM201636 block PI(3,5)P2 production; furthermore, the effect of these inhibitors on endosomal size in HeLa cells, and the effect of Apilimod on PtdInsP production in HT1080 WT and Diaskedin KO cells are shown.
**Figure S4.** The effect of AMPK and ERK inhibition on the formation of characteristic enlarged endosomes in HT1080 cells.
**Figure S5.** A subcellular fractionation of HeLa WT cells, where VPS34IN and YM201636 inhibitors were used and a model for the proposed BORC‐AMPK‐PIKfyve interaction.

**Movie S1.** DQBSA uptake and lysosomal movement in WT and Diaskedin KO HT1080 cells.

**Movie S2.** HeLa WT and Diaskedin KO cells, stably expressing LAMP1 NeonGreen, employing live cell STED imaging.

**Movie S3.** A selected magnification, taken from Movie S2

**Movie S4.** An electron tomographic reconstruction and modeling of tubule forming late endosomal compartments in HT1080 WT cells upon 2h YM201636 inhibition and subsequent washout.

**Movie S5.** An electron tomographic reconstruction and modeling of tubule forming late endosomal compartments in HT1080 Diaskedin KO cells upon 2h YM201636 inhibition and subsequent washout.

## References

[1] Huotari J , Helenius A . Focus review endosome maturation. EMBO J. 2011;30(17):3481‐3500. 10.1038/emboj.2011.286. 21878991 PMC3181477

[2] Pu J , Schindler C , Jia R , Jarnik M , Backlund P , Bonifacino JS . BORC, a multisubunit complex that regulates lysosome positioning. Dev Cell. 2015;33(2):176‐188. 10.1016/j.devcel.2015.02.011. 25898167 PMC4788105

[3] Pu J , Guardia CM , Keren‐Kaplan T , Bonifacino JS . Mechanisms and functions of lysosome positioning. J Cell Sci. 2016;129:4329‐4339. 10.1242/jcs.196287. 27799357 PMC5201012

[4] Matteoni R , Kreis TE . Translocation and clustering of endosomes and lysosomes depends on microtubules. J Cell Biol. 1987;105(3):1253‐1265. 10.1083/jcb.105.3.1253. 3308906 PMC2114818

[5] Korolchuk VI , Saiki S , Lichtenberg M , et al. Lysosomal positioning coordinates cellular nutrient responses. Nat Cell Biol. 2011;13(4):453‐462. 10.1038/ncb2204. 21394080 PMC3071334

[6] Dozynkiewicz MA , Jamieson NB , MacPherson I , et al. Rab25 and CLIC3 collaborate to promote integrin recycling from late endosomes/lysosomes and drive cancer progression. Dev Cell. 2012;22(1):131‐145. 10.1016/j.devcel.2011.11.008. 22197222 PMC3507630

[7] Schiefermeier N , Scheffler JM , de Araujo MEG , et al. The late endosomal p14‐MP1 (LAMTOR2/3) complex regulates focal adhesion dynamics during cell migration. J Cell Biol. 2014;205:525‐540. 10.1083/jcb.201310043. 24841562 PMC4033770

[8] Falcón‐Pérez JM , Starcevic M , Gautam R , Dell'Angelica EC . BLOC‐1, a novel complex containing the pallidin and muted proteins involved in the biogenesis of melanosomes and platelet‐dense granules. J Biol Chem. 2002;277(31):28191‐28199. 10.1074/jbc.M204011200. 12019270

[9] Moriyama K , Bonifacino JS . Pallidin is a component of a multi‐protein complex involved in the biogenesis of lysosome‐related organelles. Traffic. 2002;3(9):666‐677. 10.1034/j.1600-0854.2002.30908.x. 12191018

[10] Starcevic M , Dell'Angelica EC . Identification of Snapin and three novel proteins (BLOS1, BLOS2, and BLOS3/reduced pigmentation) as subunits of biogenesis of lysosome‐related organelles complex‐1 (BLOC‐1). J Biol Chem. 2004;279(27):28393‐28401. 10.1074/jbc.M402513200. 15102850

[11] Lee HH , Nemecek D , Schindler C , et al. Assembly and architecture of biogenesis of lysosome‐related organelles complex‐1 (BLOC‐1). J Biol Chem. 2012;287(8):5882‐5890. 10.1074/jbc.M111.325746. 22203680 PMC3285357

[12] Delevoye C , Heiligenstein X , Ripoll L , et al. Bloc‐1 brings together the Actin and microtubule cytoskeletons to generate recycling endosomes. Curr Biol. 2016;26(1):1‐13. 10.1016/j.cub.2015.11.020. 26725201 PMC4713302

[13] Guardia CM , Farías GG , Jia R , Pu J , Bonifacino JS . BORC functions upstream of kinesins 1 and 3 to coordinate regional movement of lysosomes along different microtubule tracks. Cell Rep. 2016;17(8):1950‐1961. 10.1016/j.celrep.2016.10.062. 27851960 PMC5136296

[14] Filipek PA , de Araujo MEG , Vogel GF , et al. LAM TOR/Ragulator is a negative regulator of Arl8band BORC‐dependent late endosomal positioning. J Cell Biol. 2017;216(12):4199‐4215. 10.1083/jcb.201703061. 28993467 PMC5716276

[15] Pu J , Keren‐Kaplan T , Bonifacino JS . A Ragulator–BORC interaction controls lysosome positioning in response to amino acid availability. J Cell Biol. 2017;216(12):4183‐4197. 10.1083/jcb.201703094. 28993468 PMC5716277

[16] Rosa‐Ferreira C , Munro S . Arl8 and SKIP act together to link lysosomes to kinesin‐1. Dev Cell. 2011;21(6):1171‐1178. 10.1016/j.devcel.2011.10.007. 22172677 PMC3240744

[17] Bissig C , Hurbain I , Raposo G , van Niel G . PIKfyve activity regulates reformation of terminal storage lysosomes from endolysosomes. Traffic. 2017;18(11):747‐757. 10.1111/tra.12525. 28857423

[18] Choy CH , Saffi G , Gray MA , et al. Lysosome enlargement during inhibition of the lipid kinase PIKfyve proceeds through lysosome coalescence. J Cell Sci. 2018;131(10):jcs213587 10.1242/jcs.213587. 29661845 PMC6031331

[19] Zhang C‐S , Hawley SA , Zong Y , et al. Fructose‐1,6‐bisphosphate and aldolase mediate glucose sensing by AMPK. Nature. 2017;548(7665):112‐116. 10.1038/nature23275. 28723898 PMC5544942

[20] Zhang YL , Guo H , Zhang CS , et al. AMP as a low‐energy charge signal autonomously initiates assembly of axin‐ampk‐lkb1 complex for AMPK activation. Cell Metab. 2013;18(4):546‐555. 10.1016/j.cmet.2013.09.005. 24093678

[21] Borner GHH , Hein MY , Hirst J , Edgar JR , Mann M , Robinson MS . Fractionation profiling: a fast and versatile approach for mapping vesicle proteomes and protein‐protein interactions. Mol Biol Cell. 2014;25(20):3178‐3194. 10.1091/mbc.e14-07-1198. 25165137 PMC4196868

[22] Zheng N , Zhang X , Rosania GR . Effect of phospholipidosis on the cellular pharmacokinetics of chloroquine. J Pharmacol Exp Ther. 2011;336(3):661‐671. 10.1124/jpet.110.175679. 21156819 PMC3061524

[23] Min SH , Suzuki A , Stalker TJ , et al. Loss of PIKfyve in platelets causes a lysosomal disease leading to inflammation and thrombosis in mice. Nat Commun. 2014;5:4691 10.1038/ncomms5691. 25178411 PMC4369914

[24] Zhang Y . The Roles and Regulation of Phosphatidylinositol 3, 5‐Bisphosphates in Mammals [PhD Dissertation]. University of Iowa. 2008 10.17077/etd.ln4xxgq1, DOI: 10.17077/etd.ln4xxgq1

[25] Jin N , Lang MJ , Weisman LS . Phosphatidylinositol 3,5‐bisphosphate: regulation of cellular events in space and time. Biochem Soc Trans. 2016;44(1):177‐184. 10.1042/BST20150174. 26862203 PMC4836390

[26] Teis D , Huber LA . The odd couple: signal transduction and endocytosis. Cell Mol Life Sci. 2003;60(10):2020‐2033. 10.1007/s00018-003-3010-2. 14618253 PMC11138817

[27] Teis D , Taub N , Kurzbauer R , et al. p14‐MP1‐MEK1 signaling regulates endosomal traffic and cellular proliferation during tissue homeostasis. J Cell Biol. 2006;175(6):861‐868. 10.1083/jcb.200607025. 17178906 PMC2064696

[28] Sancak Y , Bar‐Peled L , Zoncu R , Markhard AL , Nada S , Sabatini DM . Ragulator‐rag complex targets mTORC1 to the lysosomal surface and is necessary for its activation by amino acids. Cell. 2010;141(2):290‐303. 10.1016/j.cell.2010.02.024. 20381137 PMC3024592

[29] Rebsamen M , Pochini L , Stasyk T , et al. SLC38A9 is a component of the lysosomal amino acid sensing machinery that controls mTORC1. Nature. 2015;519(7544):477‐481. 10.1038/nature14107. 25561175 PMC4376665

[30] Zhang C‐S , Jiang B , Li M , et al. The lysosomal v‐ATPase‐ragulator complex is a common activator for AMPK and mTORC1, acting as a switch between catabolism and anabolism. Cell Metab. 2014;20(3):526‐540. 10.1016/j.cmet.2014.06.014. 25002183

[31] de Araujo MEG , Naschberger A , Fürnrohr BG , et al. Crystal structure of the human lysosomal mTORC1 scaffold complex and its impact on signaling. Science. 2017;358(6361):377‐381. 10.1126/science.aao1583. 28935770

[32] Liu Y , Lai Y‐C , Hill EV , et al. Phosphatidylinositol 3‐phosphate 5‐kinase (PIKfyve) is an AMPK target participating in contraction‐stimulated glucose uptake in skeletal muscle. Biochem J. 2013;455(2):195‐206. 10.1042/BJ20130644. 23905686

[33] Hill EV , Hudson CA , Vertommen D , Rider MH , Tavaré JM . Regulation of PIKfyve phosphorylation by insulin and osmotic stress. Biochem Biophys Res Commun. 2010;397(4):650‐655. 10.1016/j.bbrc.2010.05.134. 20513353

[34] Teis D , Wunderlich W , Huber LA . Localization of the MP1‐MAPK scaffold complex to endosomes is mediated by p14 and required for signal transduction. Dev Cell. 2002;3(6):803‐814. 10.1016/S1534-5807(02)00364-7. 12479806

[35] de Araújo MEG , Stasyk T , Taub N , et al. Stability of the endosomal scaffold protein LAMTOR3 depends on heterodimer assembly and proteasomal degradation. J Biol Chem. 2013;288(25):18228‐18242. 10.1074/jbc.M112.349480. 23653355 PMC3689965

[36] Vogel GF , Ebner HL , de Araujo MEGG , et al. Ultrastructural morphometry points to a new role for LAMTOR2 in regulating the endo/lysosomal system. Traffic. 2015;16(6):617‐634. 10.1111/tra.12271. 25677580

[37] Lin SC , Hardie DG . AMPK: sensing glucose as well as cellular energy status. Cell Metab. 2018;27(2):299‐313. 10.1016/j.cmet.2017.10.009. 29153408

[38] Zong Y , Zhang CS , Li M , et al. Hierarchical activation of compartmentalized pools of AMPK depends on severity of nutrient or energy stress. Cell Res. 2019;29:460‐473. 10.1038/s41422-019-0163-6. 30948787 PMC6796943

[39] Du J , Guan T , Zhang H , Xia Y , Liu F , Zhang Y . Inhibitory crosstalk between ERK and AMPK in the growth and proliferation of cardiac fibroblasts. Biochem Biophys Res Commun. 2008;368(2):402‐407. 10.1016/j.bbrc.2008.01.099. 18243130

[40] Medina DL , Di Paola S , Peluso I , et al. Lysosomal calcium signalling regulates autophagy through calcineurin and TFEB. Nat Cell Biol. 2015;17(3):288‐299. 10.1038/ncb3114. 25720963 PMC4801004

[41] Dong X , Shen D , Wang X , et al. PI(3,5)P2 mucolipin Ca2+ controls membrane traffic by direct activation of release channels in the endolysosome. Nature. 2010;1(4):1‐21. 10.1038/ncomms1037.PI. PMC2928581 20802798

[42] Wang W , Gao Q , Yang M , et al. Up‐regulation of lysosomal TRPML1 channels is essential for lysosomal adaptation to nutrient starvation. Proc Natl Acad Sci. 2015;112(11):E1373‐E1381. 10.1073/pnas.1419669112. 25733853 PMC4371935

[43] Kilpatrick BS , Yates E , Grimm C , Schapira AH , Patel S . Endo‐lysosomal TRP mucolipin‐1 channels trigger global ER Ca 2+ release and Ca 2+ influx. J Cell Sci. 2016;129(20):3859‐3867. 10.1242/jcs.190322. 27577094 PMC5087663

[44] Klionsky DJ , Klionsky DJ , Abdelmohsen K , et al. Guidelines for the use and interpretation of assays for monitoring autophagy (3rd edition). Autophagy. 2016;12(1):1‐222. 10.1080/15548627.2015.1100356. 26799652 PMC4835977

[45] Jin N , Chow CY , Liu L , et al. VAC14 nucleates a protein complex essential for the acute interconversion of PI3P and PI(3,5)P2in yeast and mouse. EMBO J. 2008;27(24):3221‐3234. 10.1038/emboj.2008.248. 19037259 PMC2600653

[46] Botelho RJ , Efe JA , Teis D , Emr SD . Assembly of a Fab1 phosphoinositide kinase signaling complex requires the Fig 4 phosphoinositide phosphatase. Mol Biol Cell. 2008;19(10):4273‐4286. 10.1091/mbc.e08-04-0405. 18653468 PMC2555960

[47] Ikonomov OC , Sbrissa D , Fenner H , Shisheva A . PIKfyve‐ArPIKfyve‐Sac3 core complex: contact sites and their consequence for Sac3 phosphatase activity and endocytic membrane homeostasis. J Biol Chem. 2009;284(51):35794‐35806. 10.1074/jbc.M109.037515. 19840946 PMC2791009

[48] Efeyan A , Zoncu R , Chang S , et al. Regulation of mTORC1 by the Rag GTPases is necessary for neonatal autophagy and survival. Nature. 2013;493(7434):679‐683. 10.1038/nature11745. 23263183 PMC4000705

[49] Chow CY , Zhang Y , Dowling JJ , et al. Mutation of FIG 4 causes neurodegeneration in the pale tremor mouse and patients with CMT4J. Nature. 2007;448(7149):68‐72. 10.1038/nature05876. 17572665 PMC2271033

[50] Chow CY , Landers JE , Bergren SK , et al. Deleterious variants of FIG 4, a phosphoinositide phosphatase, in patients with ALS. Am J Hum Genet. 2009;84(1):85‐88. 10.1016/j.ajhg.2008.12.010. 19118816 PMC2668033

[51] Ferguson CJ , Lenk GM , Meisler MH . Defective autophagy in neurons and astrocytes from mice deficient in PI(3,5)P2. Hum Mol Genet. 2009;18(24):4868‐4878. 10.1093/hmg/ddp460. 19793721 PMC2778378

[52] Shalem O , Sanjana NE , Hartenian E , et al. Genome‐scale CRISPR‐Cas9 knockout screening in human cells. Science. 2014;343(6166):84‐87. 10.1126/science.1247005. 24336571 PMC4089965

[53] Hsu PD , Scott DA , Weinstein JA , et al. DNA targeting specificity of RNA‐guided Cas9 nucleases. Nat Biotechnol. 2013;31(9):827‐832. 10.1038/nbt.2647. 23873081 PMC3969858

[54] Yu C , Zhang Y , Yao S , Wei Y . A PCR based protocol for detecting indel mutations induced by TALENs and CRISPR/Cas9 in zebrafish. PLoS ONE. 2014;9(6):e98282 10.1371/journal.pone.0098282. 24901507 PMC4046980

[55] Swift S , Lorens J , Achacoso P , Nolan GP . Rapid production of retroviruses for efficient gene delivery to mammalian cells using 293T cell‐based systems Current Protocols in Immunology. New York, NY: John Wiley & Sons, Inc.; 2001: 10.17.14‐10.17.29. 10.1002/0471142735.im1017cs31. 18432682

[56] Hess MW , Vogel GF , Yordanov TE , et al. Combining high‐pressure freezing with pre‐embedding immunogold electron microscopy and tomography. Traffic. 2018;19(8):639‐649. 10.1111/tra.12575. 29673018

[57] Studer D , Michel M , Müller M . High pressure freezing comes of age. Scanning Microsc Suppl. 1989;3:253‐269. 2694271

[58] Liou W , Geuze HJ , Slot JW . Improving structural integrity of cryosections for immunogold labeling. Histochem Cell Biol. 1996;106(1):41‐58. 10.1007/BF02473201. 8858366

[59] Yu L , McPhee CK , Zheng L , et al. Termination of autophagy and reformation of lysosomes regulated by mTOR. Nature. 2010;465(7300):942‐946. 10.1038/nature09076. 20526321 PMC2920749

[60] Ho CY , Choy CH , Botelho RJ . Radiolabeling and quantification of cellular Levels of phosphoinositides by high performance liquid chromatography‐coupled flow Scintillation. J Vis Exp. 2016;(107):e53529 10.3791/53529. PMC4781033 26780479

