# Supplementary material for: Biogenesis of lysosome‐related organelles complex‐1 (BORC) regulates late endosomal/lysosomal size through PIKfyve‐dependent phosphatidylinositol‐3,5‐bisphosphate
Source: Traffic. 2019 Aug 19;20(9):674–96. doi: 10.1111/tra.12679 (PMC6771566; doi:10.1111/tra.12679)
Supplement: Supplementary file 1 — Figure S1. An overview of the endosomal system of HT1080 WT cells, labeling of oversized endocytic compartments in HT1080 WT cells with lysosomal markers and a subcellular fractionation of BORC deletion mutants. Figure S2. Effect of Chloroquine and a selection of other chemical inhibitors on the frequency of the characteristic enlarged late endosomes in BORC deletion mutants in HT1080. Figure S3. Schematically where VPS34IN and YM201636 block PI(3,5)P2 production; furthermore, the effect of these inhibitors on endosomal size in HeLa cells, and the effect of Apilimod on PtdInsP production in HT1080 WT and Diaskedin KO cells are shown. Figure S4. The effect of AMPK and ERK inhibition on the formation of characteristic enlarged endosomes in HT1080 cells. Figure S5. A subcellular fractionation of HeLa WT cells, where VPS34IN and YM201636 inhibitors were used and a model for the proposed BORC‐AMPK‐PIKfyve interaction. [file TRA-20-674-s001.docx]

**BORC regulates late endosomal/lysosomal size through PIKfyve-dependent phosphatidylinositol-3,5-bisphosphate**

# Authors

Teodor E. Yordanov^1^, Victoria E. B. Hipolito^2^, Gudrun Liebscher^1^, Georg F. Vogel^1,4^, Taras Stasyk^1^, Caroline Herrmann^1^, Stephan Geley^5^, David Teis^1^, Roberto J. Botelho^2^, Michael W. Hess^3*^, Lukas A. Huber^1,6*^

^1^Division of Cell Biology, Biocenter, Medical University of Innsbruck, Innsbruck, Austria

^2^Department of Chemistry and Biology and the Graduate Program in Molecular Science, Ryerson University, Toronto, Ontario, Canada

**^3^**Division of Histology and Embryology, Medical University of Innsbruck, Innsbruck, Austria

^4^Department of Pediatrics I, Medical University of Innsbruck, Innsbruck, Austria

^5^Division of Molecular Pathophysiology, Biocenter, Medical University of Innsbruck, Innsbruck, Austria

^6^Austrian Drug Screening Institute, ADSI, Innsbruck, Austria

*Correspondence (equally contributing corresponding authors):

Lukas A. Huber, orcid.org/0000-0003-1116-2120, [lukas.a.huber@i-med.ac.at](mailto:lukas.a.huber@i-med.ac.at), Biocenter, Cell Biology, Medical University of Innsbruck, Innrain 80-82, 6020 Innsbruck, Austria

Michael W. Hess, orcid.org/0000-0002-5154-3553, [michael.hess@i-med.ac.at](mailto:michael.hess@i-med.ac.at), Division of Histology and Embryology, Medical University of Innsbruck, Müllerstrasse 59, 6020 Innsbruck,

Austria

**Supplemental Materials**

## Supplementary Figure S1. Characterization of HT1080 cells.

(A) Confocal images of WT HT1080 cells, stained with the late endosomal markers LAMP1 and CD63, the early endosomal marker EEA1 or with Mannose 6-phosphate receptor (M6PR). Scale bars: 10um. (B) LAMP1 detection on the enlarged late endosomes in HT1080 WT cells at steady state through means of cryobased pre-embedding immunogold electron microscopy^56^. Label can be seen both at the limiting membrane (arrows) and inside the organelles. Scale bar: 500nm. (C) Cathepsin D immunogold label on the same enlarged late endosomes in Diaskedin KO cells, stably re-expressing WT HA-tagged Diaskedin (see also Figure 2A HT1080 Rescue); standard Tokuyasu-cryosection labelling technique. Scale bar: 500nm. (D) Representative WB of a subcellular fractionation of WT, Diaskedin KO, Myrlysin KO and Lyspersin KO HT1080 cells that were fractionated in PNS, Cytosol and TM. (E) Quantification of the subcellular fractionation, depicting the percentage of TM-bound amount of the indicated protein in each of the genotypes. Data presented as mean values ± standard deviation. Unpaired Student’s t test was performed between all genotypes (*p≤0.05; **p≤0.01; ***p≤0.001).

##

## Supplementary Figure S2. Deletion of Diaskedin and Myrlysin but not Lyspersin renders characteristic enlarged late endosomes in HT1080 less sensitive to lysosomal inhibition.

1. IF of WT, Diaskedin KO, Myrlysin KO and Lyspersin KO HT1080 cells, treated with 50uM Chloroquine (CQ) for 2h and stained with LAMP1 (green) and EEA1 (red). Scale bars: 10um.
2. Quantification of the percentage of cells that display HT1080 characteristic enlarged late endosomes either untreated in steady state or upon Chloroquine treatment (CQ). In parallel to steady state cells (refer to Figure 3B); CQ-treated cells were counted from at least 3 biological replicates with over 100 cells per genotype pro replica. Data presented as mean values ± standard deviation. Unpaired Student’s t test was performed for every genotype in the two tested conditions (*p≤0.05; **p≤0.01; ***p≤0.001). (C) Quantification of the percentage of cells that display HT1080 characteristic enlarged late endosomes upon treatment with various inhibitors or upon amino acid (AA) starvation. Cells were counted from at least 3 biological replicates with over 100 cells per genotype and tested condition pro replica. Data presented as mean values ± standard deviation. Unpaired Student’s t test was performed between each condition tested for every corresponding genotype (*p≤0.05; **p≤0.01; ***p≤0.001).

## Supplementary Figure S3. Inhibition of PI(3,5)P_2_ synthesis induces the formation of enlarged late endosomes in HeLa cells.

(A) Schematic representation of where the indicated inhibitors block PI(3,5)P2 synthesis in the biosynthetic pathway. (B) Super resolution STED images of WT and Diaskedin KO HeLa cells in steady state, treated with either 2.5µM VPS34IN or 800nM YM201636 for 1h or upon 1h washout of the indicated inhibitor (Recovery) and stained with LAMP1. Scale bars: 1um. (C) Diameter of late endosomes in WT and Diaskedin KO HeLa cells. Manual size measurement based on at least 100 endosomes from at least 6 cells counted in each of the indicated conditions. Data presented as median value with the box representing the 25^th^ and 75^th^ percentile, and the whiskers- the minimal and maximal values. Unpaired Student’s t test was performed between WT and Diaskedin KO cells in each of the indicated conditions (*p≤0.05; **p≤0.01; ***p≤0.001). (D) WT and Diaskedin KO HT1080 cells were incubated with ^3^H-*myo*- inositol and the indicated PtdInsP species and their abundancy was compared in either steady state or upon Apilimod addition. Data presented as normalized (to total PI) mean values ± standard deviation. Unpaired Student’s t test was performed between WT and Diaskedin KO for every PtdIns species where a difference of over 1.5x fold (dotted line) was observed from at least three independent biological replicates (*p≤0.05; **p≤0.01; ***p≤0.001).

##

## Supplementary Figure S4. Blocking of AMPK but not MAPK signaling induces the formation of characteristic enlarged late endosomes in HT1080 cells.

(A) Representative WB of WT and Diaskedin KO HT1080 cell lysates at steady state, upon the addition of 2%BSA for 24h to the full media, upon 24h FBS starvation or upon the addition of 100nM EGF for 1h. (B) IF of WT and Diaskedin KO HT1080 cells in steady state or treated with either 100nM PD0325901 overnight or 10µM Dorsomorphin for 3h and stained with LAMP1 (green) and EEA1 (red). Scale bars: 10µm. (C) Quantification of the percentage of cells that display HT1080 characteristic enlarged late endosomes in steady state and upon overnight 100nM PD0325901 or 3h 10µM Dorsomorphin treatment. Cells were counted from at least 3 biological replicates with over 100 cells per genotype and tested condition pro replica. Data presented as mean values ± standard deviation. Unpaired Student’s t test was performed between each genotype either in steady state or upon overnight 100nM PD0325901 or 3h 10µM Dorsomorphin treatment (*p≤0.05; **p≤0.01; ***p≤0.001). (D) Representative WB of WT and Diaskedin KO HT1080 cells in steady state and upon overnight 100nM PD0325901 or 3h 10µM Dorsomorphin treatment.

##

## Supplementary Figure S5. Inhibition of PI(3,5)P_2_ synthesis leads to relocalization of the BORC complex.

(A) Representative WB of subcellular fractionation of HeLa WT cells in steady state or treated with either 2.5µM VPS34IN or 800nM YM201636 for 6h that were fractionated in Cytosol and total membrane (TM) fractions. (B) Quantification of the amount of Diaskedin and Lyspersin present on TM fractions in steady state or upon VPS34IN or YM201636 addition. Data presented as mean values ± standard deviation. Unpaired Student’s t test was performed between all tested conditions per protein of interest (*p≤0.05; **p≤0.01; ***p≤0.001). (C) Model of PI(3,5)P_2_ regulation. Components of the BORC complex, namely Diaskedin and the anchor Myrlysin act to suppress PIKfyve activation by AMPK. Upon removal of these components, AMPK can hyper-activate PIKfyve, which leads to increased PI(3,5)P_2_ production and induces tubule formation.

## Supplementary Movie S1

DQBSA uptake and lysosomal movement in WT and Diaskedin KO HT1080 cells. Cells were fed with 10µg/ml DQBSA for 12h and analyzed by time-lapse epifluorescence microscopy. Frames were taken for 3min at 1s intervals.

## Supplementary Movie S2

HeLa WT and Diaskedin KO cells, stably expressing LAMP1 NeonGreen were imaged over a period of 23.5s with a super resolution STED microscope. The arrow indicates the endosome depicted in Fig. 5. Scale bars: 1µm.

## Supplementary Movie S3

Individual endosomes from HeLa WT and Diaskedin KO, selected from Movie S2.

## Supplementary Movie S4

Electron tomographic reconstructions of tubule forming late endosomal compartments in HT1080 WT cells upon 2h YM201636 inhibition and subsequent washout. Intraluminal vesicles (ILVs) are shown in yellow, the endosomes’ limiting membrane in red.

## Supplementary Movie S5

Electron tomographic reconstructions of tubule forming late endosomal compartments in HT1080 Diaskedin KO cells upon 2h YM201636 inhibition and subsequent washout. Intraluminal vesicles (ILVs) are shown in yellow, the endosomes’ limiting membrane in red.
